# Is England closing the international gap in cancer survival?

**DOI:** 10.1038/bjc.2015.265

**Published:** 2015-08-04

**Authors:** Sarah Walters, Sara Benitez-Majano, Patrick Muller, Michel P Coleman, Claudia Allemani, John Butler, Mick Peake, Marianne Grønlie Guren, Bengt Glimelius, Stefan Bergström, Lars Påhlman, Bernard Rachet

**Affiliations:** 1 Cancer Survival Group, London School of Hygiene and Tropical Medicine, Keppel Street, London WC1E 7HT, UK; 2 Department of Gynaecological Oncology, Royal Marsden Hospital, London SW3 6JJ, UK; 3 Glenfield Hospital, University Hospitals of Leicester, Groby Road, Leicester LE3 9QP, UK; 4 Department of Oncology, Oslo University Hospital, Ullevaal, PO Box 4956, Nydalen, NO-0424 Oslo, Norway; 5 K. G. Jebsen Colorectal Cancer Research Centre, Oslo University Hospital, PO Box 4953, Nydalen, NO-0424 Oslo, Norway; 6 Department of Immunology, Genetics and Pathology, Uppsala University, Akademiska sjukhuset, SE-751 85 Uppsala, Sweden; 7 Department of Oncology, Gävle Hospital, SE-801 87 Gävle, Sweden; 8 Department of Surgical Sciences, Uppsala University, Akademiska sjukhuset, SE-751 85 Uppsala, Sweden

**Keywords:** cancer survival, international comparison, population-based, cancer registries

## Abstract

**Background:**

We provide an up-to-date international comparison of cancer survival, assessing whether England is ‘closing the gap' compared with other high-income countries.

**Methods:**

Net survival was estimated using national, population-based, cancer registrations for 1.9 million patients diagnosed with a cancer of the stomach, colon, rectum, lung, breast (women) or ovary in England during 1995–2012. Trends during 1995–2009 were compared with estimates for Australia, Canada, Denmark, Norway and Sweden. Clinicians were interviewed to help interpret trends.

**Results:**

Survival from all cancers remained lower in England than in Australia, Canada, Norway and Sweden by 2005–2009. For some cancers, survival improved more in England than in other countries between 1995–1999 and 2005–2009; for example, 1-year survival from stomach, rectal, lung, breast and ovarian cancers improved more than in Australia and Canada. There has been acceleration in lung cancer survival improvement in England recently, with average annual improvement in 1-year survival rising to 2% during 2010–2012. Survival improved more in Denmark than in England for rectal and lung cancers between 1995–1999 and 2005–2009.

**Conclusions:**

Survival has increased in England since the mid-1990s in the context of strategic reform in cancer control, however, survival remains lower than in comparable developed countries and continued investment is needed to close the international survival gap.

The gap in cancer survival between England and comparable countries has galvanised policymakers and clinicians since EUROCARE first launched its European survival comparisons (Berrino *et al*, 1995). Evidence that survival is generally lower in England has led to target setting and increased investment, aiming to raise survival in England to the standards achieved elsewhere.

Since the Calman–Hine Report recommended strategic improvements to cancer services in England, there has been a series of policy initiatives to improve survival (Expert Advisory Group on Cancer, 1995). The NHS Cancer Plan for England (Department of Health, 2000) was the second national cancer plan in the world (following Norway's). It emphasised centralisation, specialisation and use of multi-disciplinary teams (MDTs). A further suite of measures to improve prevention, earlier diagnosis and patient management was launched through the Cancer Reform Strategy (Department of Health, 2007) to address continuing concerns about the survival deficit in comparison to other high-income countries.

In 2009, the Department of Health in England formed the International Cancer Benchmarking Partnership (ICBP), a consortium of epidemiologists, clinicians and policymakers tasked with understanding survival differences between the United Kingdom and five other high-income countries with universal healthcare system coverage: Australia, Canada, Denmark, Norway and Sweden. The ICBP demonstrated steady improvement in survival from colorectal, lung, breast and ovarian cancers in all six countries for patients diagnosed during 1995–2007, but showed that survival in the United Kingdom and Denmark was consistently lower than elsewhere, and there was no evidence of ‘catch-up' with the other countries, with the exception of breast cancer (Coleman *et al*, 2011).

‘Closing the gap' therefore remains an on-going focus for UK health policy. At the time of publication of a new national cancer strategy for England, this paper uses the most up-to-date data available to ask whether England is now closing the international gap in cancer survival. Given the influence of the ICBP in defining the recent cancer policy agenda in England, we include the same cancers and countries as that study, with the addition of stomach cancer. We assess progress in a variety of ways, considering both the absolute ‘gap' and relative improvement over time.

## Materials and Methods

### Patients

Individual cancer registrations for 1.9 million adults (aged 15–99 years) diagnosed with a cancer of the stomach, colon, rectum, lung, breast (women) or ovary in England during 1995–2012, and followed up to 31 December 2013 were obtained from the National Cancer Registry at the Office for National Statistics, linked to the National Health Service Central Register for information on the eventual death of these patients. Cancer registration is a live process, with continual updating of historic registrations, and the current data set was extracted on 22 May 2014.

One- and five-year survival estimates for a further 1.9 million patients diagnosed with one of these six cancers in Australia, Canada, Denmark, Norway or Sweden during 1995–2009, and followed up to 31 December 2009, were extracted from the CONCORD-2 study (Allemani *et al*, 2015). The CONCORD programme, based at the London School of Hygiene and Tropical Medicine, conducts global surveillance of cancer survival using data from population-based cancer registries. The CONCORD-2 study comprised analysis of individual data for 25.7 million adults (aged 15–99 years) diagnosed with any of ten cancers, and 75 000 children diagnosed with childhood acute lymphoblastic leukaemia, from 279 cancer registries in 67 countries.

Whereas the ICBP data were national for Denmark, Norway and the United Kingdom, here the coverage is national for England, Sweden (cf. 43% coverage in ICBP), Canada (cf. 65%), Denmark and Norway, and 91% for Australia (cf. 60%).

The same inclusion criteria were applied to the National Cancer Registry data for England as to the CONCORD-2 data (Allemani *et al*, 2015). Patients diagnosed with an invasive, primary, malignant neoplasm of one of the specified sites were eligible for analysis, and they were excluded if their tumour was benign (not malignant) or *in situ* (malignant but not invasive) or of uncertain behaviour (uncertain whether benign or malignant), or if the organ of origin was unknown. If a patient was diagnosed with two invasive primary tumours at different sites, both tumours were included. If a patient was diagnosed with two primary tumours at the same site, only the first tumour was considered. Patients whose tumour was registered through death certification only were excluded (data quality control statistics by cancer site in Supplementary Webtables 1–6).

The CONCORD-2 topographical definition of tumour sites was applied using the International Classification of Diseases for Oncology (3rd Edition; ICD-O-3). This diverges slightly from the specification used in the production of National Statistics on cancer in England (details available on request; Table 1).

### Methods

**Figure 1:**
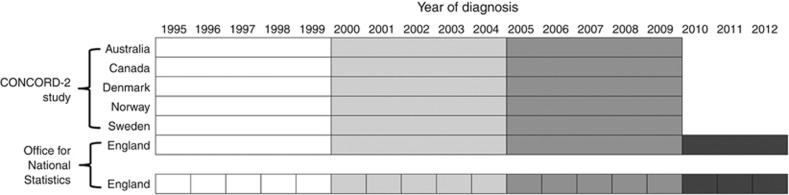
**Data sources and periods of diagnosis.** The data in our study originated either from the CONCORD-2 study or from the Office for National Statistics. Patients from England were grouped based on their date of diagnosis both by calendar periods of the CONCORD-2 study (for comparability with the other countries) and by year (to allow analysis of yearly changes).

Methods and tools used in the CONCORD-2 study were applied to the more up-to-date National Cancer Registry data for England in order to compare cancer survival in all six countries for the calendar periods of diagnosis 1995–1999, 2000–2004 and 2005–2009. Survival was also estimated for patients diagnosed during 2010–2012 in England, as well as year-on-year trends for 1995–2012, with follow-up to 2013 (Figure 1).

Cancer patients can die from causes other than their cancer. We estimated net survival, which represents survival in the hypothetical scenario that the cancer in question is the only possible cause of death, factoring out mortality from other causes. This background mortality was estimated using life tables of all-cause mortality rates in the population. We used the same life tables for England as were used in the CONCORD-2 analyses, which were specific to single year of age, calendar year, sex and Government Office Region. To estimate net survival, we used the Stata tool *stns* (Clerc-Urmès *et al*, 2014), which implements the Pohar–Perme estimator (Pohar Perme *et al*, 2012). It takes into account how competing risks of death from other causes increase with age.

One- and five-year net survival were estimated using the traditional cohort method for the periods of diagnosis 1995–1999 and 2000–2004. To estimate 1- and 5-year net survival using a cohort approach, it is necessary to have the potential of 1 or 5 full years of follow-up, respectively, for each patient, which was not the case for patients diagnosed in 2005–2009 in the CONCORD-2 study. A period approach was therefore used for these patients (Brenner and Gefeller, 1996). For comparability, we also used period analysis for the calendar period 2005–2009 in England, even though we did have enough follow-up data to estimate 1-year survival for these patients. For patients diagnosed in England in 2010–2012, we used cohort analysis to estimate 1-year net survival, and a period approach was needed to estimate 5-year net survival (see Supplementary Webfigures 1 and 2 for structure of survival analyses for 1-year and 5-year survival by calendar period).

Year-on-year trends in England for patients diagnosed during 1995–2012, with follow-up to the end of 2013 were estimated using a cohort approach for 1-year net survival. Cohort analysis was also used to estimate 5-year net survival for patients diagnosed up to 2008, and a hybrid approach was used to predict survival at 5 years in 2013 (Brenner and Rachet, 2004) (see Supplementary Webfigures 3 and 4 for structure of year-on-year analyses of 1-year and 5-year survival).

Net survival from cancer varies by age, so apparent differences in overall survival between two cancer patient populations can be driven by underlying differences in the age distributions of those populations. To limit this potential bias, we present age-standardised estimates of net survival, using the same age weights as employed in the CONCORD-2 analyses to enable comparison. These were the International Cancer Survival Standard (ICSS) weights, for which age at diagnosis was categorised into five groups: 15–44 years, 45–54 years, 55–64 years, 65–74 years and 75–99 years (Corazziari *et al*, 2004).

To assess whether or not England had ‘closed the gap' in cancer survival, we interrogated the results in three ways. First, we assessed whether survival for patients diagnosed in 2005–2009 in England equalled or exceeded that in the other countries: where survival was higher in England, or where the 95% confidence interval for the difference between the two-point estimates included zero, we considered England to have ‘caught up' with the other country. Second, we asked whether survival in England by 2010–2012 had ‘caught up' with the level observed in each other country in 2005–2009. Third, we assessed whether survival in England had improved more than elsewhere during 1995–2009, even if ‘catch-up' had not yet been achieved, by considering the confidence intervals for the difference in improvement between England and each other country. To do this, we calculated how much survival had improved in each other country between 1995–1999 and 2005–2009 and compared this to the amount of improvement in England. We report on these differences with reference to their 95% confidence intervals; the difference in improvements between England and the other country was considered statistically significant at the 5% level if the confidence interval for the difference did not include zero.

Finally, we evaluated the year-on-year trend in England up to 2012, examining whether there had been any acceleration in improvement in survival in more recent years. Using the year-on-year estimates for England for patients diagnosed during 1995–2012, we calculated the annual change in survival during the periods 1995–1999, 2000–2004, 2005–2009 and 2010–2012. This was the total percentage change in survival (e.g., the arithmetic difference between survival in 1995 and 1999) divided by the number of annual increments (e.g., the period 1995–1999 includes 5 years, but four annual increments).

We identified a panel of 57 clinicians with expertise in one of the six cancers, using purposive sampling of the international clinical networks of senior colleagues in cancer registries and research institutions, ensuring a roughly balanced distribution by country and cancer. We used the Delphi approach to establish consensus of opinion between participants (Dalkey and Helmer, 1963; Hsu and Sandford, 2007). Clinicians were first invited to respond to a semi-structured questionnaire before seeing the results of the survival analyses, to elicit their perceptions of how and why survival at the population level might have changed in their country during 1995–2009. We then distributed the survival analyses and the broad hypotheses arising from the questionnaires, and we conducted in-depth interviews (face to face and by telephone) with individual clinicians. We received responses from 25 clinicians (including 16 questionnaires and 16 interviews), fairly representative of cancers and countries.

## Results

### Years of diagnosis 1995–2009

Net survival was highest for patients diagnosed with breast cancer, in the range 94–97% 1 year after diagnosis and 81–86% at 5 years for those diagnosed in 2005–2009. Survival was lowest from lung cancer, in the range 31–42% at 1 year and 10–17% at 5 years in 2005–2009 (Tables 2 and 3).

One- and five-year survival from the gastrointestinal cancers was highest in Australia, and for the gynaecological cancers survival was highest in Australia, Norway and Sweden. Lung cancer survival was highest in Australia, Canada and Sweden. Survival point estimates were slightly lower in England than in Denmark for all cancer sites at 1 and 5 years after diagnosis, with the exceptions of stomach cancer (whole study period) and rectal cancer (in 1995–1999).

There was improvement in 1- and 5-year survival over time for all cancers and in all countries. The mean improvement in 1-year survival among the six participating countries between 1995–1999 and 2005–2009 was 2% for breast cancer, and 5–7% for the other five cancers. For 5-year survival, the mean improvement was 6–7% for colon and rectal cancers, and 3-4% for the other four cancers.

**Figure 2:**
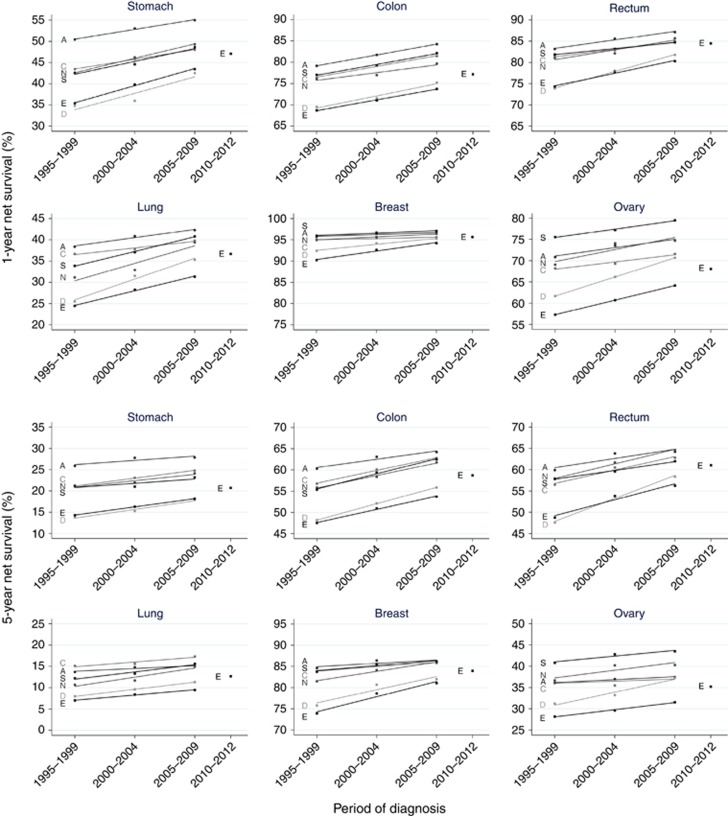
**Trends in 1- and 5-year net survival in Australia (A), Canada (C), Denmark (D), England (E), Norway (N) and Sweden (S) by period of diagnosis.** Estimates of net survival are presented for the calendar periods of diagnosis 1995–1999, 2000–2004 and 2005–2009. Simple linear regression lines are presented for each combination of country and cancer using data from these three periods, to indicate the average change in survival. An estimate of net survival for England only is also presented for the calendar period of diagnosis 2010–2012.

One- and five-year survival were lowest in England and Denmark for all cancers, and, with few exceptions, remained so throughout 1995–2009 (Figure 2). The exceptions were that by 2005–2009 there was no evidence of difference between Denmark and Canada in 1-year survival from breast and ovarian cancers, and by that time, there was also no evidence of difference in 5-year survival from ovarian cancer between Denmark and either Australia or Canada. By contrast, there is evidence that survival in England during 2005–2009 remained lower than in Australia, Canada, Norway and Sweden for all six cancers.

### Calendar period of diagnosis 2010–2012 in England *vs* 2005–2009 in other countries

Survival for patients diagnosed with one of these six cancers in England in 2010–2012 generally remained lower than for the equivalent patients diagnosed in 2005–2009 in Australia, Canada, Norway and Sweden (Tables 2 and 3). The exceptions were that there was no evidence of difference in 1-year survival for patients diagnosed with a cancer of the stomach, rectum or breast in 2010–2012 in England compared with patients diagnosed with one of those cancers in 2005–2009 in Canada. Similarly, there was no evidence of difference in 1-year survival for patients diagnosed with stomach cancer in 2010–2012 in England compared with patients diagnosed in 2005–2009 in Norway or Sweden, or for rectal cancer patients in England compared with Sweden at 1 or 5 years after diagnosis.

### Trends in cancer survival in England compared with Australia, Canada, Norway and Sweden, 1995–2009

For patients diagnosed in 1995–1999, the average gap in 1-year survival between England and the four leading countries was 5–14% by 2005–2009 it was 2–11%. Equivalent figures for 5-year survival were 6–10% and 5–9%, respectively. Although England had not closed the gap in survival with Australia, Canada, Norway or Sweden by 2005–2009, the difference between the improvement in England and the improvement in each other country (improvement in England minus other country improvement) was generally positive (Tables 2 and 3). There was evidence at the 5% level of statistical significance that the improvement in 1-year survival between 1995–1999 and 2005–2009 in England was greater than in Australia and Canada for patients diagnosed with any of these cancers except for cancer of the colon. In addition, there was evidence that the improvement was greater for 1-year survival compared with Norway for colon and breast cancers, and compared with Sweden for rectal, breast and ovarian cancers.

There was also evidence that the improvement in 5-year survival between 1995–1999 and 2005–2009 was greater in England than in Australia for all but ovarian cancer. In addition, the improvement in 5-year survival from rectal cancer was greater than in Sweden, and for ovarian cancer compared with Canada. The improvement in 1- and 5-year survival from breast cancer was greater than in all four leading countries.

### Trends in cancer survival in England compared with Denmark, 1995–2009

Survival in Denmark was generally as low as in England in 1995–1999, though significantly slightly higher for lung, breast and ovarian cancer at 1 and 5 years (Tables 2 and 3, Figure 2). The difference in the improvement in England compared with the improvement in Denmark between 1995–1999 and 2005–2009 was however generally negative, that is, survival improved more in Denmark than in England for these cancers, except for breast cancer at 1 and 5 years, and stomach cancer at 1 year. There is evidence at the 5% level of statistical significance that survival improved more in Denmark than in England at 1 and 5 years for rectal cancer, and at 1 year for lung cancer. For patients diagnosed in 2005–2009, survival was lower in England than in Denmark for colon and rectal cancers at 1 and 5 years, as well as remaining lower for lung, breast (1-year survival only) and ovarian cancers. One-year survival from lung cancer in Denmark was 1.1% (0.3–1.8%) higher than in England in 1995–1999, but by 2005–2009, survival was 4.0% (3.2–4.8%) higher in Denmark (arithmetic gap in 1- and 5-year survival: Supplementary Webtables 7 and 8).

### Survival trends in England during 1995–2012

**Figure 3:**
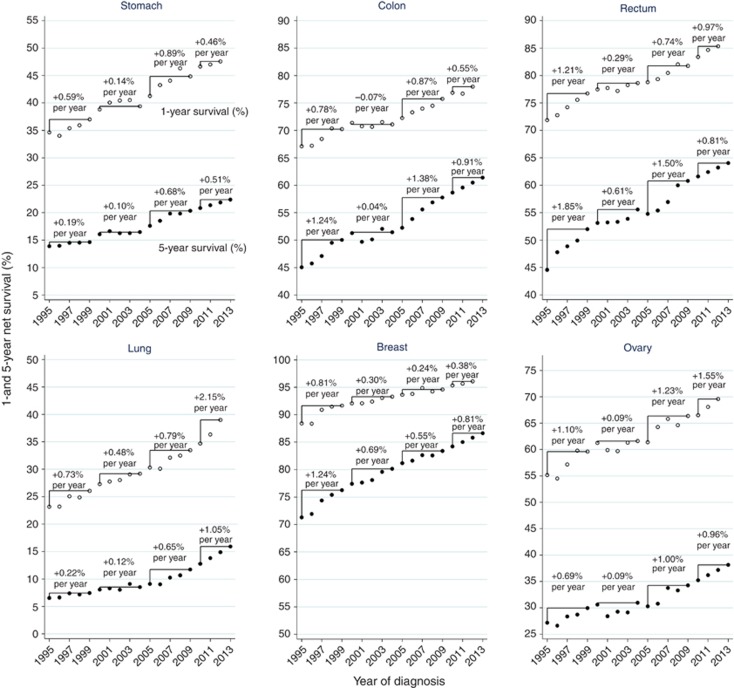
**Trends in 1- and 5-year net survival in England by year of diagnosis.** Unfilled and filled markers represent estimates of 1- and 5-year net survival, respectively. The average arithmetic improvement in survival between years is shown for the calendar periods 1995–1999, 2000–2004, 2005–2009 and 2010–2013 above the trends.

The year-on-year trends in survival in England during 1995–2012 show a period of relative improvement in net survival in the late 1990s, followed by some years of stability in the early 2000s (Figure 3). During 2005–2009, survival again improved more quickly particularly for 1- and 5-year survival from colon, rectal and ovarian cancers. For stomach and lung cancers, that pattern can be seen for 1-year survival but less so for 5-year survival, which showed rather little annual improvement before 2005, followed by steady improvement, especially for lung cancer. Breast cancer is the exception: the biggest annual improvements in 1- and 5-year survival occurred in the late 1990s, with smaller but steady improvements since then, particularly for 5-year survival. For lung cancer, 1-year survival has improved more rapidly since 2007/2008, with the annual improvement peaking at 2.2% per annum for patients diagnosed in 2010–2012. Five-year lung cancer survival also improved, but more slowly, at 1.1% per annum from 2010.

## Discussion

Despite steady improvement in survival from stomach, colon, rectal, lung, breast and ovarian cancers in England over the past two decades, survival remained lower than in Australia, Canada, Norway and Sweden for patients diagnosed in 2005–2009, and typically also for patients diagnosed in 2010–2012 in England compared with those diagnosed in 2005–2009 elsewhere. The improvement in survival between 1995–1999 and 2005–2009 was sometimes larger than in the leading countries, particularly in comparison with Australia, Canada and Sweden, leading to some narrowing of the international cancer survival gap. As in England, Denmark also had relatively low survival in comparison to the other four countries in 1995–1999, and cancer control has similarly engendered considerable public debate in that country. Survival generally improved more in Denmark than in England between 1995–1999 and 2005–2009, particularly for lung and rectal cancers. Since 2009, improvement in survival from lung cancer has accelerated in England, and there has also been slight acceleration for ovarian, breast and rectal cancers.

### Explaining trends in England

In England, survival generally rose in the late 1990s, followed by a relatively stable period in the early 2000s, after which it improved more quickly, apart from for breast cancer where the greatest gains were seen in the 1990s followed by continued but smaller annual increments throughout the 2000s. Relative stability in survival in the early 2000s was previously identified in an evaluation of the impact of the NHS Cancer Plan for England (Rachet *et al*, 2009). That strategy, launched in September 2000, proposed to inject an additional £570 million into cancer services by 2003–2004 to improve prevention, screening, diagnosis and treatment; it established Cancer Networks to oversee specialisation and centralisation of cancer care, following the earlier recommendations of Calman and Hine (Department of Health, 2000). Rachet *et al* (2009) found that despite the increased investment in services, survival in England did not increase during 2001–2003 as much as in Wales, which had not yet implemented a national cancer plan. These trends were reversed during 2004–2006, once the new structures and relationships in England were properly established (House of Commons Committee of Public Accounts, 2006).

In order to fully evaluate the impact of the NHS Cancer Plan on survival, the need for data up to the end of 2009 was noted (Rachet *et al*, 2009). We present those data here, showing that accelerating improvement in survival in England did arise in the mid-2000s. There is consensus among clinicians and auditors that this improvement can be attributed to the national reforms launched during 1995–2000 and their continuing momentum throughout the 2000s, manifest in two revisions to the national strategy and growing emphasis on outcomes monitoring (Department of Health, 2007, 2011). Increased investment, specialisation and centralisation, greater use of MDTs, target-monitoring and performance review are described as key drivers of change in clinical culture and practice, and as the main reasons for improved cancer patient outcomes in England in the 2000s (House of Commons Committee of Public Accounts, 2011).

Treatment in high-volume, specialised centres by sub-specialists with high caseloads is associated with provision of more appropriate treatment and better patient outcomes (Birkmeyer *et al*, 2003; Hannan *et al*, 2002; Meyer, 2005), and the drive to increase such centralisation had an impact in England. For example, during 2000–2009, the percentage of women with ovarian cancer receiving surgery who were treated in a specialist trust rose from 43 to 76%, and the number treated by a specialist surgeon (caseload of >18 patients per year) rose from 20 to 55% (Butler *et al*, 2015). For lung cancer, the number of specialist thoracic surgeons doubled from 44 in 2006 to 84 whole-time equivalent (WTE) in 2014 (personal communication: Sridhar Rathinam, Workforce Lead for the Society of Cardiothoracic Surgeons, 28 April 2015), meaning that more resections were performed by lung specialists, rather than cardiothoracic generalists as in the past (Page *et al*, 2011). The total number of resections for lung cancer carried out in the United Kingdom and Ireland each year has risen from *c*.3000 in 2001–2002 to *c.*5000 by 2009–2010 (Page *et al*, 2011), and to over 6000 by 2011–2012 (Society for Cardiothoracic Surgery in Great Britain and Ireland, undated), and the overall percentage of patients resected in England and Wales rose from 9 to 15% during 2005–2013 (Health and Social Care Information Centre, 2014; The NHS Information Centre, 2006). At the same time, increased specialisation has meant that surgeons are more willing to operate on patients with higher risks of poor outcomes, such as those with co-morbidities or older patients, so, for example, there has been a particularly large increase in the resection of lung cancer patients older than 65 years (Riaz *et al*, 2012).

Specialisation and improved diagnostic investigations have led to better targeting of treatment and lower postoperative mortality. For example, 30-day mortality following gastrectomy was 4.5% in 2007–2009 in England (Cromwell *et al*, 2010), compared with 12.0% in 24 hospitals in England and Wales in 1999–2002 (McCulloch *et al*, 2003). It is likely this is partly due to the increase in the percentage of specialist centres that have three or more specialist surgeons (as per the guidelines) from 53% in 2007 to 95% in 2012, and partly because by 2012 nearly all stomach cancer patients eligible for curative treatment were seen by a specialist MDT (Groene *et al*, 2014). Thirty-day postoperative mortality from colorectal cancer also declined from 6.8% in 1998 to 5.8% in 2006, with the biggest decline occurring in 2005–2006 (Morris *et al*, 2011).

Reforms in cancer services in England have contributed to improved survival over the past two decades for these six cancers, but these changes did not necessarily lead to a closed gap in survival between England and other countries, because despite relative improvement over time, there may remain comparative shortfalls in provision, partly due to the implementation of parallel reforms elsewhere.

### Comparison of cancer control reforms

Both Norway and Denmark had launched national cancer plans by 2000, which similarly emphasised centralisation, specialisation and improved patient pathways (Danish National Board of Health, 2000; Norwegian Official Reports, 1997). The early EUROCARE studies had a similar effect in Denmark as they did in England, showing lower survival than in comparable European nations, and prompting public and parliamentary scrutiny of Danish cancer control (Olesen *et al*, 2009). The Danish cancer plans that were published throughout the 2000s had strong political support and a wide mandate, leading to rapid implementation. Since 2001, surgery for lung cancer has been concentrated from seven departments to four (Starr *et al*, 2013), ovarian cancer surgery was reduced from 47 departments to 8 during 2004–2007 (Ottesen *et al*, 2009) and gastric cancer surgery was restricted from 37 departments to 4 university departments during 1999–2007 (Jensen *et al*, 2007; Jensen *et al*, 2010). There has also been emphasis on centralisation for colorectal cancer surgery to a greater degree than in England, although this could be because hospitals in England generally treat a larger volume of patients than in Denmark. While in England most hospital trusts conduct surgery for colorectal cancer, in Denmark the number of departments performing surgery for colon and rectal cancers reduced from 38 to 15 during 2003–2012 (Lykke *et al*, 2013).

One- and five-year survival improved more in Denmark than in England during 1995–1999 to 2005–2009 for all but stomach (1-year) and breast cancer (1- and 5-year), with statistical evidence that improvement was greater in Denmark for rectal (1- and 5-year survival) and lung cancer (1-year). This could be because of more comprehensive reform in Denmark or because strategies were better targeted. For example, both England and Denmark identified improving early diagnosis as a priority (Olesen *et al*, 2009; Richards, 2009), and this may have had more impact in Denmark, where the stage distribution was initially more adverse than in England for some cancers (Maringe *et al*, 2012; Maringe *et al*, 2013). Acknowledging the positive impact of these reforms, health policymakers and clinicians in Norway and Sweden are increasingly influenced by Denmark in designing their own cancer strategies, as shown by recent policy in both of those countries to emulate the Danish model of standardised patient pathways (Probst *et al*, 2012).

Evidence that survival in England improved faster than in other countries during 1995–2009 was strongest in relation to Australia, Canada and Sweden. This could be explained by a partial ‘ceiling effect' in those countries, given that they generally had the highest survival at the beginning of the study period. Alternatively, more regionalised health systems in those three countries might have limited the efficacy of national cancer control strategy and the power of national guidelines. While Denmark, England and Norway all introduced national cancer plans by 2000, in the federal countries of Australia and Canada the first provincial cancer plans were launched in the mid-2000s (Cancer Care Ontario, 2004; Cancer Institute New South Wales, 2004), and it was not until 2006–2007 that national oversight bodies were founded to coordinate cancer policy in the various regions. The Canadian Strategy for Cancer Control was published in 2006, leading to the founding of the Canadian Partnership Against Cancer in 2007 (Canadian Strategy for Cancer Control Governing Council, 2006). Cancer Australia was founded in 2006 and has issued a national cancer strategy for 2014–2019. Federalism as well as the remoteness of some rural communities in these countries may pose a larger challenge to the implementation of national standards than in the geographically smaller European countries, possibly explaining the slightly flatter trends in survival for some cancers, such as ovarian (5-year) and lung, despite overall higher survival (Hegney *et al*, 2005; Tracey *et al*, 2014). The first Swedish National Cancer Strategy was launched in 2009, and it is as yet unclear how successful it has been in influencing coordinated policy between the Regional Health Boards.

Colorectal oncologists in Sweden and Norway, and lung cancer specialists in England, identified the creation of national quality registries as having been instrumental in the drive towards better care and in improved outcomes in those countries. Population-based quality registries for rectal cancer were established in Norway and Sweden in 1993 and 1995, respectively, to monitor the rollout of novel surgical techniques and the impact on recurrence and survival (Påhlman *et al*, 2007; Wibe *et al*, 2002). The registries were expanded to include colon cancer in 2007 (Guren *et al*, 2015; Kodeda *et al*, 2013). Similarly, in England, the national lung cancer audit was initiated in 2004 and now includes all lung cancer patients referred to an MDT in England (Khakwani *et al*, 2013a; Rich *et al*, 2011c). Information from these quality registries is regularly fed back to cancer centres and hospital trusts, through public reports, oral presentations and meetings.

Studies have shown that the availability of these data has improved standards, persuading smaller centres to stop operating or to recruit specialist colorectal surgeons in Sweden and Norway, and leading to an increase in histological confirmation for lung cancer in England (Beckett *et al*, 2012; Påhlman *et al*, 2007). The data sets have spawned a wealth of research that should lead to better-targeted cancer-control policies (Dahlberg *et al*, 1998; Guren *et al*, 2015; Hansen *et al*, 2007; Rich *et al*, 2011a, 2011b), and they have been acknowledged as setting the standard for quality registration in Europe (Blum *et al*, 2014). Other national audits are increasingly available in England, and if these data streams continue to be accessible for research and local-level benchmarking, the prospects for continued harmonisation towards best practice are strong.

### Innovations in diagnosis and treatment

In addition to structural reforms, investment and monitoring of outcomes, improvements in survival have also been driven by innovations in diagnostics and treatment over the past two decades. Broadly speaking, innovations have been universally implemented in all six countries, but there are important differences in the timing and degree of provision, which could have contributed to differential survival in the 2000s.

In the case of colorectal cancer, major innovations that could have influenced population-based survival during 1995–2009 include improvements in preoperative staging through advances in high-resolution radiology, the uptake of total mesorectal excision (TME) surgery, the increased use of preoperative (chemo)radiotherapy and the treatment of metastatic disease. There is evidence that implementation of some of these innovations was slower in England than elsewhere. The use of advanced imaging tools for rectal cancer staging (MRI or, in Australia, positron emission tomography (PET) combined with computed tomography (CT)) has increased in most of these countries since the late 1990s. By 2011–2012, 86% of rectal cancer patients in England had an MRI before treatment (Scott *et al*, 2013: p. 69), compared with 97% of patients in Sweden receiving MRI plus investigation for metastases in the liver and lungs in 2013 (Regionalt Cancercentrum Norr, 2014). TME was pioneered in England in the early 1980s, and shown to reduce locoregional recurrence for rectal cancer. However, TME only became common practice in England much later than in the Nordic countries, where more effective efforts were made to ensure and monitor implementation (e.g., through the Norwegian Rectal Cancer Project and its equivalent in Sweden from 1993/1994) (Guren *et al*, 2015; Martling *et al*, 2005; Richards, 2009; Wibe *et al*, 2002). Although surgery has improved in England, the resection rate remains lower than it was in the early 2000s in some other countries. For example, we estimated that 32% of rectal cancer patients diagnosed in England in 2012–2013 (Scott *et al*, 2014) had an anterior resection compared with 45% in Sweden in 1995–2004 (Jung *et al* 2009) (details of calculation available on request). Similarly, for stomach cancer, while in 2000–2005 in Ontario 45% of patients were treated with curative intent, in 2011–2012 in England 38% received surgery (Coburn *et al*, 2010; Taylor *et al*, 2014: p. 5).

In relation to lung cancer, although there has been a significant increase in resection rate and resection numbers in England, and an increase in the proportion of patients seen by specialists, there remains scope for improvement. The number of thoracic surgeons almost doubled to 84 WTE during 2006–2014 (personal communication: Sridhar Rathinam, Workforce Lead for the Society of Cardiothoracic Surgeons, 28 April 2015), but with over 30 000 lung cancer patients diagnosed each year, the number remains relatively small. There is evidence that diagnostic procedures may be less aggressive in England than elsewhere. The National Lung Cancer Audit in England set a target (which has been met since 2009) of 75% for pathological confirmation of lung cancer (Health and Social Care Information Centre, 2012). In contrast in Sweden, 95% of lung cancer patients diagnosed during 2002–2010 had their tumour verified by cytology or histology (Regionalt Cancercentrum Uppsala Örebro, 2012: p. 10, Table 3). There remains wide variation in the proportion of pathological confirmation by age group and region in England: for patients aged over 75, this is just 50% (Khakwani *et al*, 2013b). In a pan-European study on the quality of lung cancer care, only 4% of participating hospitals in England reported a histological confirmation rate higher than 90%, compared with 87% of Danish participating hospitals (Blum *et al*, 2014).

These contrasts may reflect differences in clinical guidelines or access to diagnostic investigations. PET scan, usually in combination with CT, is important in the assessment of the likelihood of mediastinal and/or extra-thoracic metastasis that would contraindicate treatment with curative intent. A PET–CT positive result may represent malignant infiltration but may also be a false positive for benign inflammation, therefore, histological investigation (biopsy by ultrasound-guided needle aspiration or mediastinoscopy) may be needed to confirm malignant disease (Schmidt-Hansen *et al*, 2014). In England, PET–CT is currently only recommended for patients potentially suitable for treatment with curative intent, while in Ontario PET–CT is recommended, if available, for all patients after a pathological diagnosis of NSCLC (Cancer Care Ontario, 2012). According to English guidelines, histological investigation of mediastinal lymph nodes is only indicated as an alternative to PET–CT (National Institute for Health and Clinical Excellence (NICE), 2011), whereas in Ontario and Denmark histological confirmation is recommended together with PET–CT, for all patients with centrally located tumours or with suspected mediastinal node involvement (Dansk Lunge Cancer Gruppe, 2014; Darling *et al*, 2010).

The international trend in breast cancer survival is unusual, in that the largest improvement in survival in all countries was experienced in the 1990s, and the 2000s have been characterised by a closing survival gap between England and Denmark and the four leading countries. This is probably because although there have been continued improvements in breast cancer diagnosis and treatment (including the introduction of targeted therapies, and improved usage of antihormonal treatments and chemotherapy), major innovations for breast cancer, such as adjuvant chemotherapy and tamoxifen, were introduced in the 1970s–1980s, and population screening had also been introduced in England before our study period. Furthermore, improvements in service organisation (e.g., use of MDTs and issuance of clinical guidelines) occurred earlier than for the other five cancers in England. By 1995, 1-year survival from breast cancer was already 95% or above in the four leading countries, and it is likely that the closing international survival gap arises mainly because of a ‘ceiling effect' elsewhere.

### Recent trends and future prospects

There was an acceleration in survival improvement in England in more recent years. The average annual improvement in survival was higher in 2005–2009 than in 2000–2004 for the gastrointestinal, lung and ovarian cancers, and higher in 2010–2012 than in 2005–2009 for rectal and ovarian cancers at 1 year, and lung cancer at 1 and 5 years. The recent acceleration in lung cancer survival improvement has been especially large, and the improvement in 5-year survival since 2006 has particular significance given the relatively static trend until then. This improvement coincides with the doubling of the resection rate for lung cancer in recent years (Page *et al*, 2011).

It is unclear whether these trends represent further closing of the international survival gap because data are not yet available for the comparator countries. However, there are indications that some novel therapies and approaches, which could have affected recent survival trends in the six countries, have been implemented later in England than elsewhere. For example, the introduction of stereotactic body radiotherapy (SBRT) as an alternative to surgery for early-stage lung cancer patients could have improved lung cancer survival at the population level, given that it widens access to treatment with curative intent to patients with contraindications for surgery (Haasbeek *et al*, 2012). Data are not yet available on the relative availability of SBRT in the six countries, but it was pioneered in Sweden (Lax *et al*, 1994), it was indicated in clinical guidelines in Denmark, Ontario and Sweden earlier than in England, and it has been available in some Danish and Swedish cancer centres from the early-mid-2000s (Jeppesen *et al*, 2013; Louie *et al*, 2014). Similarly, it is unclear how far there is variation between these countries in the implementation of ultra-radical surgery for ovarian cancer, and in the introduction of perioperative chemotherapy (Cunningham *et al*, 2006) and improvements in palliative oncology for stomach cancer (Bang *et al*, 2010). The international survival gap may narrow as England introduces innovations that are already implemented in other countries, or widen as such innovations become widespread in other countries first.

Equally, there is potential for a widening survival gap if reforms implemented earlier in England are now replicated elsewhere. For example, a national bowel cancer screening programme was introduced in England earlier than elsewhere (2006–2009), possibly contributing to some of the recent improvement in bowel cancer survival. However, all five other countries have now also implemented either national or regional bowel screening programmes, which often cover a wider age range than in England. Therefore, it is likely that if improvements in stage distribution or survival were effected by screening in England, they will also soon be effected elsewhere (Flitcroft *et al*, 2010; Zavoral *et al*, 2009).

Likewise, health system factors and national cancer policy were instrumental in driving improvements in cancer outcomes in England in the 2000s, but concerns have arisen about continuity of those system-level effects following reforms implemented since through the Health and Social Care Act 2012 (Cancer Research UK, 2012; National Audit Office, 2015). Since April 2013, commissioning services for cancer have been restructured, changing from 152 Primary Care Trusts to 211 Clinical Commissioning Groups; the 28 Cancer Networks, which worked to ensure consistency and quality in cancer services for more than a decade, have been disbanded, and key strategic policy posts within the Department of Health have been changed to advisory roles within NHS England, or dissolved. These reforms came at a time of financial austerity: the health service is under pressure to make unprecedented efficiency savings of £20 billion by 2014–2015, and the average annual spend on cancer services per head of the population fell by 3.4% in 2010–2011 (Cancer Research UK, 2012). Such financial restrictions compound existing lower levels of cancer spending compared with other northwest European countries (Luengo-Fernandez *et al*, 2013).

There is already evidence that cancer services have been affected, with some performance targets having been missed for the first time (e.g., the target of a maximum 62-day wait between urgent GP referral and treatment for 85% of patients was not met during 2014–2015) (NHS England, 2015). Furthermore, although there is continued emphasis on outcomes monitoring in the reformed NHS, and cancer intelligence continues to improve, systems of formal accountability have been reshaped, implying risks to the continued dissemination and use of this intelligence to improve standards. This is compounded by increased restrictions on data access for research due to rising sensitivity around patient confidentiality (National Audit Office, 2015).

England's future trajectory towards closing the international gap in cancer survival is therefore uncertain, and continued international cancer survival surveillance is needed to monitor on-going progress. It will be important that such surveillance is age-specific, given evidence of the particularly wide gap in survival for older patients (Maringe *et al*, 2013; Walters *et al*, 2013)

## Conclusion

One- and five-year survival from six common cancers in England was lower than in Australia, Canada, Norway and Sweden in 2005–2009. By 2010-2012 1-year survival in England remained lower than in 2005–2009 in the four leading countries for three of these cancers, and 5-year survival remained lower for five of these cancers. During 1995–1999 to 2005–2009, improvement in 1-year survival was generally higher in England than in Australia, Canada or Sweden. For breast cancer, survival at both 1 and 5 years in England improved more than in these countries or Norway. There has been notable acceleration in improvement in survival from lung cancer during 2010–2012, as well some acceleration for rectal and ovarian cancers.

The improvement in survival in England was partly due to innovations in diagnostics and treatment, although there is evidence that novel therapies were often implemented more quickly elsewhere. Other key drivers were strong strategic leadership, increased investment, and the creation of more stable and centralised commissioning structures to enhance observance of national guidelines, as well as increased availability of data for outcomes monitoring and local benchmarking. Survival in Denmark was similarly lower than in the other four countries in 1995–1999, but it generally improved faster than in England over the next decade, especially for rectal and lung cancers. This may be ascribed to particularly effective centralisation, specialisation and performance monitoring in Denmark in the 2000s.

In some instances, survival in England improved more than in Australia, Canada, Norway or Sweden during the 2000s, but future trends are uncertain in the context of health service reform and efficiency savings. Committed investment in centralised, specialised and accountable services for cancer patients will be essential for continued progress against the international survival gap.

## Figures and Tables

**Table 1:** Number of patients available for analysis by calendar period of diagnosis, cancer site and country

	**Stomach**	**Colon**^a^	**Rectum**^b^	**Lung**^c^	**Breast**	**Ovary**^d^	**All cancers**
**Cancer and ICD-O-3 topography code**	**C16.0-C16.6, C16.8-C16.9**	**C18.0-C18.9, C19.9**	**C20, C21.0-C21.2, C21.8**	**C34.0-C34.3, C34.8-C34.9**	**C50.0-C50.6, C50.8-C50.9**	**C48.0-C48.2, C56.9, C57.0-C57.4, C57.7-C57.9**	
**Australia**							
1995–1999	8293	37 303	13 695	35 186	47 487	5485	147 449
2000–2004	8643	41 803	16 498	38 751	55 000	6188	166 883
2005–2009	6885	35 672	13 959	34 088	46 146	5226	141 976
1995–2009	23 821	114 778	44 152	108 025	148 633	16 899	456 308
**Canada**							
1995–1999	14 218	55 969	12 538	92 176	86 819	7253	268 973
2000–2004	14 555	65 354	15 421	101 386	95 308	7717	299 741
2005–2009	15 223	73 480	21 374	112 161	104 046	10 904	337 188
1995–2009	43 996	194 803	49 333	305 723	286 173	25 874	905 902
**Denmark**							
1995–1999	2690	11 195	5827	16 919	17 202	3182	57 015
2000–2004	2521	11 986	6407	18 664	19 323	3104	62 005
2005–2009	2803	13 487	7535	20 796	22 610	3042	70 273
1995–2009	8014	36 668	19 769	56 379	59 135	9328	189 293
**England**							
1995–1999	38 477	91 784	41 547	143 113	153 356	24 771	493 048
2000–2004	34 795	96 411	43 872	144 621	170 152	25 955	515 806
2005–2009	30 693	108 276	47 620	155 612	184 247	26 336	552 784
2010–2012	16 953	70 283	31 063	101 539	116 315	16 194	352 347
1995–2012	120 918	366 754	164 102	544 885	624 070	93 256	1 913 985
**Norway**							
1995–1999	3324	10 101	5035	9466	11 647	2497	42 070
2000–2004	2881	11 268	5377	10 881	13 339	2627	46 373
2005–2009	2560	12 440	5428	12 398	13 665	2536	49 027
1995–2009	8765	33 809	15 840	32 745	38 651	7660	137 470
**Sweden**							
1995–1999	5760	15 658	9209	13 678	27 653	4486	76 444
2000–2004	5097	16 570	9843	15 491	30 760	4376	82 137
2005–2009	4463	18 494	10 397	17 575	31 755	4137	86 821
1995–2009	15 320	50 722	29 449	46 744	90 168	12 999	245 402
**Total**							
1995–2012	220 834	797 534	322 645	1 094 501	1 246 830	166 016	3 848 360

a Includes rectosigmoid junction.

b Includes anus and anal canal.

c Excludes trachea.

d Includes fallopian tube, uterine ligaments, other and unspecified female organs, peritoneum and retroperitoneum.

Numbers of patients from other countries are equal to those in the CONCORD-2 study. The number of patients in England differs from the number in England in CONCORD-2 even for comparable calendar periods because they are from a more up-to-date extract from a live data set.

**Table 2:** Age-standardised 1-year net survival with 95% confidence intervals for adults (aged 15–99 years) by calendar period of diagnosis, country and cancer site, and 1995–2009 survival improvement in England minus survival improvement in other countries

	**1995–1999**	**2000–2004**	**2005–2009**	**2010–2012**	**1995–2009 improvement in England minus 1995–2009 improvement in other country**
**Stomach**					
Australia	50.4 (49.3, 51.5)	53.0 (52.0, 54.1)	55.0 (53.8, 56.2)		3.5 (1.6, 5.3)
Canada	43.5 (42.6, 44.3)	45.8 (45.0, 46.7)	48.0 (47.2, 48.8)		3.5 (2.1, 5.0)
Denmark	34.8 (32.9, 36.7)	36.0 (34.0, 37.9)	42.5 (40.7, 44.4)		0.3 (−2.5, 3.1)
England	35.4 (34.9, 35.9)	39.8 (39.3, 40.4)	43.5 (42.8, 44.1)	47.1 (46.3, 47.9)	
Norway	42.5 (40.6, 44.4)	46.2 (44.2, 48.2)	49.3 (47.2, 51.4)		1.3 (−1.7, 4.2)
Sweden	42.6 (41.2, 44.0)	44.6 (43.1, 46.1)	48.6 (47.0, 50.2)		2.1 (−0.2, 4.3)
**Colon**					
Australia	79.2 (78.7, 79.6)	81.7 (81.3, 82.1)	84.3 (83.9, 84.7)		0.0 (−0.8, 0.7)
Canada	76.4 (76.0, 76.8)	79.2 (78.9, 79.6)	81.4 (81.1, 81.7)		0.0 (−0.6, 0.7)
Denmark	69.5 (68.6, 70.4)	71.6 (70.7, 72.4)	75.2 (74.4, 76.0)		−0.7 (−2.0, 0.6)
England	68.7 (68.4, 69.0)	71.1 (70.8, 71.4)	73.7 (73.5, 74.0)	77.2 (76.8, 77.5)	
Norway	76.0 (75.1, 76.9)	77.0 (76.1, 77.8)	79.7 (78.9, 80.4)		1.4 (0.1, 2.7)
Sweden	77.1 (76.3, 77.8)	79.3 (78.6, 80.0)	82.1 (81.5, 82.8)		0.0 (−1.1, 1.0)
**Rectum**					
Australia	83.2 (82.5, 83.8)	85.6 (85.0, 86.2)	87.2 (86.6, 87.8)		2.0 (0.9, 3.1)
Canada	81.4 (80.7, 82.2)	82.8 (82.2, 83.5)	84.9 (84.4, 85.4)		2.5 (1.4, 3.6)
Denmark	73.9 (72.7, 75.1)	78.1 (77.0, 79.1)	81.8 (80.9, 82.7)		−1.9 (−3.5, −0.3)
England	74.3 (73.9, 74.8)	77.8 (77.4, 78.3)	80.3 (79.9, 80.7)	84.5 (84.0, 84.9)	
Norway	81.1 (79.9, 82.2)	82.2 (81.1, 83.2)	85.7 (84.7, 86.6)		1.4 (−0.2, 3.0)
Sweden	81.9 (81.0, 82.7)	83.2 (82.4, 84.0)	84.7 (84.0, 85.5)		3.2 (1.9, 4.4)
**Lung**					
Australia	38.4 (37.8, 38.9)	40.8 (40.3, 41.4)	42.3 (41.7, 42.8)		3.0 (2.1, 3.8)
Canada	36.7 (36.3, 37.0)	37.8 (37.5, 38.1)	39.8 (39.4, 40.1)		3.8 (3.2, 4.4)
Denmark	25.5 (24.8, 26.2)	31.5 (30.8, 32.2)	35.3 (34.6, 36.1)		−2.9 (−4.0, −1.9)
England	24.5 (24.2, 24.7)	28.3 (28.0, 28.5)	31.3 (31.1, 31.6)	36.7 (36.4, 37.0)	
Norway	31.2 (30.2, 32.1)	32.9 (31.9, 33.9)	39.4 (38.4, 40.3)		−1.4 (−2.8, 0.1)
Sweden	33.9 (33.1, 34.8)	37.1 (36.3, 37.9)	40.8 (40.0, 41.6)		0.0 (−1.2, 1.2)
**Breast**					
Australia	95.7 (95.5, 96.0)	96.4 (96.2, 96.7)	96.6 (96.4, 96.9)		3.1 (2.6, 3.6)
Canada	94.9 (94.7, 95.1)	95.4 (95.3, 95.6)	95.6 (95.4, 95.8)		3.3 (2.9, 3.7)
Denmark	92.4 (91.9, 92.9)	94.1 (93.7, 94.6)	95.2 (94.8, 95.7)		1.2 (0.5, 1.9)
England	90.2 (90.0, 90.4)	92.6 (92.4, 92.8)	94.2 (94.1, 94.4)	95.7 (95.5, 95.9)	
Norway	95.0 (94.5, 95.6)	95.7 (95.2, 96.2)	96.4 (95.9, 96.9)		2.6 (1.9, 3.4)
Sweden	96.0 (95.7, 96.3)	96.7 (96.4, 97.0)	97.1 (96.8, 97.4)		2.9 (2.4, 3.4)
**Ovary**					
Australia	70.9 (69.7, 72.1)	73.6 (72.4, 74.7)	74.8 (73.6, 75.9)		2.9 (1.0, 4.9)
Canada	68.2 (67.1, 69.3)	69.3 (68.3, 70.3)	71.6 (70.7, 72.5)		3.5 (1.7, 5.2)
Denmark	61.7 (60.0, 63.3)	66.2 (64.5, 67.8)	70.7 (69.1, 72.3)		−2.2 (−4.7, 0.4)
England	57.3 (56.5, 58.0)	60.7 (60.0, 61.4)	64.1 (63.5, 64.8)	68.1 (67.2, 68.9)	
Norway	69.1 (67.3, 70.8)	74.1 (72.4, 75.7)	74.7 (73.1, 76.4)		1.2 (−1.4, 3.8)
Sweden	75.6 (74.3, 76.8)	77.2 (76.0, 78.5)	79.5 (78.3, 80.8)		2.9 (0.8, 4.9)

**Table 3:** Age-standardised 5-year net survival with 95% confidence intervals for adults (aged 15–99 years) by calendar period of diagnosis, country and cancer site, and 1995–2009 survival improvement in England minus survival improvement in other countries

	**1995–1999**	**2000–2004**	**2005–2009**	**2010–2012**	**1995–2009 improvement in England minus 1995–2009 improvement in other country**
**Stomach**					
Australia	25.9 (24.8, 27.0)	27.8 (26.8, 28.9)	27.9 (26.7, 29.0)		1.9 (0.1, 3.6)
Canada	21.1 (20.4, 21.9)	23.1 (22.3, 23.9)	24.8 (24.0, 25.6)		0.2 (−1.1, 1.5)
Denmark	13.8 (12.3, 15.3)	15.3 (13.7, 16.9)	17.9 (16.2, 19.5)		−0.2 (−2.6, 2.1)
England	14.3 (13.9, 14.7)	16.3 (15.9, 16.8)	18.2 (17.6, 18.7)	20.7 (19.9, 21.4)	
Norway	21.1 (19.4, 22.9)	22.0 (20.2, 23.9)	24.1 (22.1, 26.1)		0.9 (−1.9, 3.6)
Sweden	21.2 (19.9, 22.5)	21.0 (19.6, 22.3)	23.2 (21.7, 24.6)		1.9 (−0.2, 3.9)
**Colon**					
Australia	60.3 (59.7, 61.0)	63.1 (62.5, 63.7)	64.2 (63.6, 64.8)		2.3 (1.2, 3.3)
Canada	56.8 (56.3, 57.3)	60.1 (59.6, 60.6)	62.8 (62.4, 63.3)		0.2 (−0.7, 1.1)
Denmark	48.2 (47.1, 49.4)	52.1 (50.9, 53.2)	55.9 (54.8, 57.0)		−1.5 (−3.2, 0.2)
England	47.5 (47.1, 48.0)	50.9 (50.5, 51.3)	53.7 (53.3, 54.1)	58.7 (58.3, 59.2)	
Norway	55.9 (54.6, 57.2)	58.4 (57.2, 59.6)	61.8 (60.6, 62.9)		0.3 (−1.5, 2.1)
Sweden	55.4 (54.4, 56.5)	59.4 (58.5, 60.4)	62.5 (61.6, 63.5)		−0.9 (−2.4, 0.6)
**Rectum**					
Australia	59.9 (58.9, 61.0)	63.8 (62.9, 64.7)	64.2 (63.3, 65.1)		3.2 (1.6, 4.8)
Canada	56.5 (55.4, 57.5)	60.5 (59.6, 61.5)	62.8 (61.9, 63.7)		1.2 (−0.4, 2.8)
Denmark	47.6 (46.0, 49.2)	53.8 (52.2, 55.3)	58.4 (56.9, 59.8)		−3.3 (−5.6, −1.0)
England	48.8 (48.1, 49.4)	53.8 (53.3, 54.4)	56.3 (55.7, 56.8)	61.0 (60.3, 61.8)	
Norway	57.8 (56.1, 59.5)	61.7 (60.1, 63.3)	64.6 (63.0, 66.2)		0.7 (−1.8, 3.2)
Sweden	57.9 (56.6, 59.2)	59.6 (58.4, 60.8)	62.0 (60.9, 63.2)		3.4 (1.5, 5.3)
**Lung**					
Australia	13.7 (13.3, 14.2)	14.8 (14.4, 15.2)	15.0 (14.6, 15.5)		1.1 (0.4, 1.8)
Canada	15.1 (14.8, 15.3)	15.6 (15.4, 15.9)	17.3 (17.1, 17.6)		0.2 (−0.2, 0.7)
Denmark	8.0 (7.5, 8.5)	9.6 (9.1, 10.1)	11.3 (10.7, 11.9)		−0.9 (−1.7, 0.0)
England	7.0 (6.9, 7.2)	8.4 (8.2, 8.6)	9.5 (9.3, 9.7)	12.7 (12.4, 13.0)	
Norway	10.7 (10.0, 11.5)	11.7 (10.9, 12.4)	15.0 (14.1, 15.8)		−1.9 (−3.0, −0.7)
Sweden	12.2 (11.6, 12.9)	13.3 (12.7, 14.0)	15.6 (14.9, 16.4)		−1.0 (−2.0, 0.1)
**Breast**					
Australia	84.6 (84.0, 85.2)	86.4 (85.9, 86.9)	86.2 (85.6, 86.8)		5.5 (4.6, 6.5)
Canada	83.7 (83.3, 84.1)	85.3 (84.9, 85.7)	85.8 (85.5, 86.2)		5.0 (4.3, 5.8)
Denmark	75.8 (74.8, 76.8)	80.7 (79.8, 81.6)	82.0 (81.1, 82.9)		0.9 (−0.5, 2.4)
England	73.9 (73.6, 74.3)	78.6 (78.3, 79.0)	81.1 (80.7, 81.4)	84.0 (83.6, 84.4)	
Norway	81.5 (80.3, 82.6)	84.1 (83.0, 85.1)	85.9 (84.9, 87.0)		2.7 (1.1, 4.4)
Sweden	83.8 (83.1, 84.5)	85.6 (84.9, 86.3)	86.2 (85.5, 86.9)		4.7 (3.6, 5.8)
**Ovary**					
Australia	36.1 (34.8, 37.4)	37.0 (35.7, 38.3)	37.5 (36.2, 38.8)		1.9 (−0.2, 4.1)
Canada	36.5 (35.3, 37.7)	35.5 (34.4, 36.6)	37.5 (36.3, 38.6)		2.3 (0.4, 4.3)
Denmark	31.2 (29.5, 33.0)	33.2 (31.5, 34.9)	37.3 (35.4, 39.2)		−2.8 (−5.6, 0.1)
England	28.2 (27.4, 29.0)	29.6 (28.9, 30.4)	31.5 (30.8, 32.3)	35.2 (34.2, 36.2)	
Norway	36.7 (34.7, 38.8)	40.2 (38.2, 42.3)	40.3 (38.3, 42.4)		−0.3 (−3.4, 2.9)
Sweden	40.8 (39.2, 42.4)	42.8 (41.2, 44.4)	43.5 (41.9, 45.1)		0.6 (−1.9, 3.2)
