# Supplementary material for: Is England closing the international gap in cancer survival?
Source: Br J Cancer. 2015 Aug 4;113(5):848–60. doi: 10.1038/bjc.2015.265 (PMC4559829; doi:10.1038/bjc.2015.265)
Supplement: Supplementary Information [file bjc2015265x1.pdf]

Supplementary Web Appendix

Web Appendix Figure 1	p2	Structure of survival analysis for one-year survival by calendar period
Web Appendix Figure 2	p3	Structure of survival analysis for five-year survival by calendar period
Web Appendix Figure 3	p4	Structure of survival analysis for one-year survival by year of diagnosis
Web Appendix Figure 4	p5	Structure of survival analysis for five-year survival by year of diagnosis
Web Appendix Table 1	p6	Data quality control by calendar period of diagnosis (records and patients ineligible and excluded) - Stomach cancer
Web Appendix Table 2	p7	Data quality control by calendar period of diagnosis (records and patients ineligible and excluded) - Colon cancer
Web Appendix Table 3	p8	Data quality control by calendar period of diagnosis (records and patients ineligible and excluded) - Rectal cancer
Web Appendix Table 4	p9	Data quality control by calendar period of diagnosis (records and patients ineligible and excluded) - Lung cancer
Web Appendix Table 5	p10	Data quality control by calendar period of diagnosis (records and patients ineligible and excluded) - Breast cancer
Web Appendix Table 6	p11	Data quality control by calendar period of diagnosis (records and patients ineligible and excluded) - Ovarian cancer
Web Appendix Table 7	p12	Arithmetic gap in one-year net survival with 95% confidence intervals for adults (aged 15-99 years) between England and each other country, by calendar period of diagnosis, country and cancer site
Web Appendix Table 8	p13	Arithmetic gap in five-year net survival with 95% confidence intervals for adults (aged 15-99 years) between England and each other country, by calendar period of diagnosis, country and cancer site

Web Appendix Figure 1. Structure of survival analysis for one-year survival by calendar period

Calendar year of follow-up (columns); Calendar year of diagnosis (rows)

Calendar year of diagnosis	1995	1996	1997	1998	1999	2000	2001	2002	2003	2004	2005	2006	2007	2008	2009	2010	2011	2012	2013	Calendar year of diagnosis
**1995**	0	1	2	3	4	5	6	7	8	9	10	11	12	13	14	15	16	17	18	**1995**
**1996**		0	1	2	3	4	5	6	7	8	9	10	11	12	13	14	15	16	17	**1996**
**1997**			0	1	2	3	4	5	6	7	8	9	10	11	12	13	14	15	16	**1997**
**1998**				0	1	2	3	4	5	6	7	8	9	10	11	12	13	14	15	**1998**
**1999**					0	1	2	3	4	5	6	7	8	9	10	11	12	13	14	**1999**
**2000**						0	1	2	3	4	5	6	7	8	9	10	11	12	13	**2000**
**2001**							0	1	2	3	4	5	6	7	8	9	10	11	12	**2001**
**2002**								0	1	2	3	4	5	6	7	8	9	10	11	**2002**
**2003**									0	1	2	3	4	5	6	7	8	9	10	**2003**
**2004**										0	1	2	3	4	5	6	7	8	9	**2004**
**2005**											0	1	2	3	4	5	6	7	8	**2005**
**2006**												0	1	2	3	4	5	6	7	**2006**
**2007**													0	1	2	3	4	5	6	**2007**
**2008**														0	1	2	3	4	5	**2008**
**2009**															0	1	2	3	4	**2009**
**2010**																0	1	2	3	**2010**
**2011**																	0	1	2	**2011**
**2012**																		0	1	**2012**

- Estimate of one-year survival for patients diagnosed during 1995-99 using the cohort approach
- Estimate of one-year survival for patients diagnosed during 2000-04 using the cohort approach
- Estimate of one-year survival for calendar period 2005-09 using the period approach
- Estimate of one-year survival for calendar period 2010-12 using the period approach

Web Appendix Figure 2. Structure of survival analysis for five-year survival by calendar period

Calendar year of follow-up (columns); Calendar year of diagnosis (rows)

	1995	1996	1997	1998	1999	2000	2001	2002	2003	2004	2005	2006	2007	2008	2009	2010	2011	2012	2013	
**1995**	0	1	2	3	4	5	6	7	8	9	10	11	12	13	14	15	16	17	18	**1995**
**1996**		0	1	2	3	4	5	6	7	8	9	10	11	12	13	14	15	16	17	**1996**
**1997**			0	1	2	3	4	5	6	7	8	9	10	11	12	13	14	15	16	**1997**
**1998**				0	1	2	3	4	5	6	7	8	9	10	11	12	13	14	15	**1998**
**1999**					0	1	2	3	4	5	6	7	8	9	10	11	12	13	14	**1999**
**2000**						0	1	2	3	4	5	6	7	8	9	10	11	12	13	**2000**
**2001**							0	1	2	3	4	5	6	7	8	9	10	11	12	**2001**
**2002**								0	1	2	3	4	5	6	7	8	9	10	11	**2002**
**2003**									0	1	2	3	4	5	6	7	8	9	10	**2003**
**2004**										0	1	2	3	4	5	6	7	8	9	**2004**
**2005**											0	1	2	3	4	5	6	7	8	**2005**
**2006**												0	1	2	3	4	5	6	7	**2006**
**2007**													0	1	2	3	4	5	6	**2007**
**2008**														0	1	2	3	4	5	**2008**
**2009**															0	1	2	3	4	**2009**
**2010**																0	1	2	3	**2010**
**2011**																	0	1	2	**2011**
**2012**																		0	1	**2012**

- Estimate of five-year survival for patients diagnosed during 1995-99 using the cohort approach
- Estimate of five-year survival for patients diagnosed during 2000-04 using the cohort approach
- Estimate of five-year survival for calendar period 2005-09 using the period approach
- Estimate of five-year survival for calendar period 2010-12 using the period approach

Web Appendix Figure 3. Structure of survival analysis for one-year survival by year of diagnosis

Calendar year of follow-up

Calendar year of diagnosis

	1995	1996	1997	1998	1999	2000	2001	2002	2003	2004	2005	2006	2007	2008	2009	2010	2011	2012	2013	
**1995**	**0**	**1**	2	3	4	5	6	7	8	9	10	11	12	13	14	15	16	17	18	**1995**
**1996**		**0**	**1**	2	3	4	5	6	7	8	9	10	11	12	13	14	15	16	17	**1996**
**1997**			**0**	**1**	2	3	4	5	6	7	8	9	10	11	12	13	14	15	16	**1997**
**1998**				**0**	**1**	2	3	4	5	6	7	8	9	10	11	12	13	14	15	**1998**
**1999**					**0**	**1**	2	3	4	5	6	7	8	9	10	11	12	13	14	**1999**
**2000**						**0**	**1**	2	3	4	5	6	7	8	9	10	11	12	13	**2000**
**2001**							**0**	**1**	2	3	4	5	6	7	8	9	10	11	12	**2001**
**2002**								**0**	**1**	2	3	4	5	6	7	8	9	10	11	**2002**
**2003**									**0**	**1**	2	3	4	5	6	7	8	9	10	**2003**
**2004**										**0**	**1**	2	3	4	5	6	7	8	9	**2004**
**2005**											**0**	**1**	2	3	4	5	6	7	8	**2005**
**2006**												**0**	**1**	2	3	4	5	6	7	**2006**
**2007**													**0**	**1**	2	3	4	5	6	**2007**
**2008**														**0**	**1**	2	3	4	5	**2008**
**2009**															**0**	**1**	2	3	4	**2009**
**2010**																**0**	**1**	2	3	**2010**
**2011**																	**0**	**1**	2	**2011**
**2012**																		**0**	**1**	**2012**

Calendar year of diagnosis

Annual estimates of one-year survival using the cohort approach

Web Appendix Figure 4. Structure of survival analysis for five-year survival by year of diagnosis *

Calendar year of follow-up (columns); Calendar year of diagnosis (rows)

Calendar year of diagnosis	1995	1996	1997	1998	1999	2000	2001	2002	2003	2004	2005	2006	2007	2008	2009	2010	2011	2012	2013	Calendar year of diagnosis
**1995**	**0**	**1**	**2**	**3**	**4**	**5**	6	7	8	9	10	11	12	13	14	15	16	17	18	**1995**
**1996**		**0**	**1**	**2**	**3**	**4**	**5**	6	7	8	9	10	11	12	13	14	15	16	17	**1996**
**1997**			**0**	**1**	**2**	**3**	**4**	**5**	6	7	8	9	10	11	12	13	14	15	16	**1997**
**1998**				**0**	**1**	**2**	**3**	**4**	**5**	6	7	8	9	10	11	12	13	14	15	**1998**
**1999**					**0**	**1**	**2**	**3**	**4**	**5**	6	7	8	9	10	11	12	13	14	**1999**
**2000**						**0**	**1**	**2**	**3**	**4**	**5**	6	7	8	9	10	11	12	13	**2000**
**2001**							**0**	**1**	**2**	**3**	**4**	**5**	6	7	8	9	10	11	12	**2001**
**2002**								**0**	**1**	**2**	**3**	**4**	**5**	6	7	8	9	10	11	**2002**
**2003**									**0**	**1**	**2**	**3**	**4**	**5**	6	7	8	9	10	**2003**
**2004**										**0**	**1**	**2**	**3**	**4**	**5**	6	7	8	9	**2004**
**2005**											**0**	**1**	**2**	**3**	**4**	**5**	6	7	8	**2005**
**2006**												**0**	**1**	**2**	**3**	**4**	**5**	6	7	**2006**
**2007**													**0**	**1**	**2**	**3**	**4**	**5**	6	**2007**
**2008**														**0**	**1**	**2**	**3**	**4**	**5**	**2008**
**2009**															0	1	2	3	**4**	**2009**
**2010**																0	1	2	**3**	**2010**
**2011**																	0	1	**2**	**2011**
**2012**																		**0**	**1**	**2012**

[boxed] Annual estimates of five-year survival using the cohort approach

[shaded] * Prediction of five-year survival for year 2013 using the hybrid approach

Web Appendix Table 1. Data quality control by calendar period of diagnosis (records and patients ineligible and excluded) - Stomach cancer

	1995-2000			2000-2004			2005-2009			2010-2012			1995-2012		
	**Records**	**Patients**	**%**	**Records**	**Patients**	**%**	**Records**	**Patients**	**%**	**Records**	**Patients**	**%**	**Records**	**Patients**	**%**
**Total registrered**	**42,370**	**42,337**	**100.0**	**37,347**	**37,314**	**100.0**	**33,470**	**33,410**	**100.0**	**18,851**	**18,807**	**100.0**	**132,038**	**131,868**	**100.0**
Incomplete data*	29	29	<0.1	14	14	<0.1	32	32	<0.1	4	4	<0.1	79	79	<0.1
Not resident in England	33	33	<0.1	6	6	<0.1	1	1	<0.1	0	0	0.0	40	40	<0.1
In situ neoplasm	224	218	0.5	310	299	0.8	467	440	1.3	321	306	1.6	1,322	1,263	1.0
Benign or uncertain	298	289	0.7	523	513	1.4	1,073	1,057	3.2	1,101	1,085	5.8	2,995	2,944	2.2
Metastatic	25	25	<0.1	25	25	<0.1	13	13	<0.1	0	0	0.0	63	63	<0.1
Kaposi Sarcoma	0	0	0.0	0	0	0.0	1	1	<0.1	1	1	<0.1	2	2	<0.1
Otherwise ineligible	0	0	0.0	0	0	0.0	0	0	0.0	0	0	0.0	0	0	0.0
Lymphoma	0	0	0.0	0	0	0.0	0	0	0.0	0	0	0.0	0	0	0.0
Leukaemia or myeloma	0	0	0.0	0	0	0.0	0	0	0.0	0	0	0.0	0	0	0.0
Aged 100+	19	19	<0.1	20	20	<0.1	20	20	<0.1	18	18	<0.1	77	77	<0.1
**Total ineligible**		**613**	**1.4**		**877**	**2.4**		**1,564**	**4.7**		**1,414**	**7.5**		**4,468**	**3.4**
**Total eligible**		**41,724**	**100.0**		**36,437**	**100.0**		**31,846**	**100.0**		**17,393**	**100.0**		**127,400**	**100.0**
Vital status unknown	91	58	0.1	105	91	0.2	82	54	0.2	46	43	0.2	324	246	0.2
Sex not known	0	0	0.0	0	0	0.0	2	0	0.0	0	0	0.0	2	0	0.0
Sex-site error	0	0	0.0	0	0	0.0	0	0	0.0	0	0	0.0	0	0	0.0
Site-morphology mismatch	37	37	<0.1	53	53	0.1	134	134	0.4	13	13	<0.1	237	237	0.2
Age-site mismatch	0	0	0.0	0	0	0.0	0	0	0.0	0	0	0.0	0	0	0.0
Age-morphology mismatch	0	0	0.0	0	0	0.0	0	0	0.0	0	0	0.0	0	0	0.0
Age-site-morphology mismatch	0	0	0.0	0	0	0.0	0	0	0.0	0	0	0.0	0	0	0.0
Invalid dates**	5	3	<0.1	5	4	<0.1	2	1	<0.1	2	1	<0.1	14	9	<0.1
Death certificate only	3,012	2,990	7.2	1,447	1,440	4.0	931	920	2.9	358	355	2.0	5,748	5,705	4.5
Duplicate registration	0	0	0.0	0	0	0.0	0	0	0.0	0	0	0.0	0	0	0.0
Synchronous tumours†	155	148	0.4	48	45	0.1	34	29	<0.1	19	14	<0.1	256	236	0.2
Multiple primary same site‡	23	11	<0.1	21	9	<0.1	28	15	<0.1	24	14	<0.1	96	49	<0.1
**Total exclusions**		**3,247**	**7.8**		**1,642**	**4.5**		**1,153**	**3.6**		**440**	**2.5**		**6,482**	**5.1**
**Patients included in analysis**		**38,477**	**92.2**		**34,795**	**95.5**		**30,693**	**96.4**		**16,953**	**97.5**		**120,918**	**94.9**

* Missing year of birth; missing month and/or year of diagnosis; missing year of last known vital status

** For example, 31 February, or date of diagnosis earlier than date of birth

† Two or more invasive primary malignancies in the same person, in the same site, and with the same date of diagnosis

‡ Two or more invasive primary malignancies in the same person, in the same site, but with different dates of diagnosis

Web Appendix Table 2. Data quality control by calendar period of diagnosis (records and patients ineligible and excluded) - Colon cancer

	1995-2000			2000-2004			2005-2009			2010-2012			1995-2012		
	**Records**	**Patients**	**%**	**Records**	**Patients**	**%**	**Records**	**Patients**	**%**	**Records**	**Patients**	**%**	**Records**	**Patients**	**%**
**Total registrered**	**103,119**	**102,483**	**100.0**	**106,988**	**106,081**	**100.0**	**122,481**	**120,893**	**100.0**	**79,884**	**78,863**	**100.0**	**412,472**	**408,320**	**0.0**
Incomplete data*	79	79	<0.1	22	22	<0.1	19	19	<0.1	4	4	<0.1	124	124	<0.1
Not resident in England	140	140	0.1	52	51	<0.1	1	1	<0.1	2	2	<0.1	195	194	<0.1
In situ neoplasm	440	419	0.4	1,446	1,381	1.3	4,463	4,223	3.5	4,327	4,056	5.1	10,676	10,079	2.5
Benign or uncertain	2,745	2,534	2.5	3,280	3,049	2.9	3,993	3,718	3.1	1,749	1,664	2.1	11,767	10,965	2.7
Metastatic	222	221	0.2	208	208	0.2	206	205	0.2	118	118	0.1	754	752	0.2
Kaposi Sarcoma	0	0	0.0	1	1	<0.1	1	1	<0.1	0	0	0.0	2	2	<0.1
Otherwise ineligible	183	23	<0.1	140	1	<0.1	172	0	0.0	115	0	0.0	610	24	<0.1
Lymphoma	0	0	0.0	0	0	0.0	0	0	0.0	0	0	0.0	0	0	0.0
Leukaemia or myeloma	0	0	0.0	0	0	0.0	0	0	0.0	0	0	0.0	0	0	0.0
Aged 100+	32	32	<0.1	39	39	<0.1	47	47	<0.1	38	38	<0.1	156	156	<0.1
**Total ineligible**		**3,448**	**3.4**		**4,752**	**4.5**		**8,214**	**6.8**		**5,882**	**7.5**		**22,296**	**5.5**
**Total eligible**		**99,035**	**100.0**		**101,329**	**100.0**		**112,679**	**100.0**		**72,981**	**100.0**		**386,024**	**100.0**
Vital status unknown	267	183	0.2	238	210	0.2	150	132	0.1	305	301	0.4	960	826	0.2
Sex not known	1	0	0.0	1	0	0.0	1	0	0.0	0	0	0.0	3	0	0.0
Sex-site error	0	0	0.0	0	0	0.0	0	0	0.0	0	0	0.0	0	0	0.0
Site-morphology mismatch	67	67	<0.1	62	62	<0.1	34	34	<0.1	37	37	<0.1	200	200	<0.1
Age-site mismatch	0	0	0.0	0	0	0.0	0	0	0.0	0	0	0.0	0	0	0.0
Age-morphology mismatch	0	0	0.0	0	0	0.0	0	0	0.0	0	0	0.0	0	0	0.0
Age-site-morphology mismatch	25	25	<0.1	25	25	<0.1	37	37	<0.1	31	31	<0.1	118	118	<0.1
Invalid dates**	11	10	<0.1	18	2	<0.1	12	6	<0.1	4	3	<0.1	45	21	<0.1
Death certificate only	5,762	5,697	5.8	3,366	3,327	3.3	2,444	2,409	2.1	1,051	1,031	1.4	12,623	12,464	3.2
Duplicate registration	0	0	0.0	0	0	0.0	0	0	0.0	0	0	0.0	0	0	0.0
Synchronous tumours†	1,006	700	0.7	882	551	0.5	1,165	646	0.6	719	397	0.5	3,772	2,294	0.6
Multiple primary same site‡	869	569	0.6	1,178	741	0.7	1,973	1,139	1.0	1,400	898	1.2	5,420	3,347	0.9
**Total exclusions**		**7,251**	**7.3**		**4,918**	**4.9**		**4,403**	**3.9**		**2,698**	**3.7**		**19,270**	**5.0**
**Patients included in analysis**		**91,784**	**92.7**		**96,411**	**95.1**		**108,276**	**96.1**		**70,283**	**96.3**		**366,754**	**95.0**

* Missing year of birth; missing month and/or year of diagnosis; missing year of last known vital status

** For example, 31 February, or date of diagnosis earlier than date of birth

† Two or more invasive primary malignancies in the same person, in the same site, and with the same date of diagnosis

‡ Two or more invasive primary malignancies in the same person, in the same site, but with different dates of diagnosis

Web Appendix Table 3. Data quality control by calendar period of diagnosis (records and patients ineligible and excluded) - Rectal cancer															
	**1995-2000**			**2000-2004**			**2005-2009**			**2010-2012**			**1995-2012**		
	**Records**	**Patients**	**%**	**Records**	**Patients**	**%**	**Records**	**Patients**	**%**	**Records**	**Patients**	**%**	**Records**	**Patients**	**%**
**Total registrered**	**47,207**	**47,043**	**100.0**	**49,646**	**49,423**	**100.0**	**54,604**	**54,388**	**100.0**	**35,533**	**35,411**	**100.0**	**226,757**	**226,032**	**100.0**
Incomplete data*	46	46	<0.1	19	19	<0.1	18	18	<0.1	10	10	<0.1	93	93	<0.1
Not resident in England	76	76	0.2	32	32	<0.1	2	1	<0.1	1	1	<0.1	111	110	<0.1
In situ neoplasm	596	575	1.2	1,352	1,306	2.6	3,378	3,294	6.1	3,173	3,083	8.7	8,499	8,258	3.7
Benign or uncertain	2,603	2,510	5.3	2,729	2,622	5.3	2,392	2,315	4.3	525	519	1.5	8,249	7,966	3.5
Metastatic	19	18	<0.1	20	20	<0.1	13	13	<0.1	1	1	<0.1	53	52	<0.1
Kaposi Sarcoma	0	0	0.0	0	0	0.0	0	0	0.0	0	0	0.0	0	0	0.0
Otherwise ineligible	0	0	0.0	0	0	0.0	0	0	0.0	0	0	0.0	0	0	0.0
Lymphoma	0	0	0.0	0	0	0.0	0	0	0.0	0	0	0.0	0	0	0.0
Leukaemia or myeloma	0	0	0.0	0	0	0.0	0	0	0.0	0	0	0.0	0	0	0.0
Aged 100+	28	28	<0.1	14	14	<0.1	34	32	<0.1	12	12	0.1	88	86	<0.1
**Total ineligible**		**3,253**	**6.9**		**4,013**	**8.1**		**5,673**	**10.4**		**3,626**	**10.2**		**16,565**	**7.3**
**Total eligible**		**43,790**	**93.1**		**45,410**	**91.9**		**48,715**	**89.6**		**31,785**	**89.8**		**169,700**	**100.0**
Vital status unknown	181	133	0.7	160	143	0.7	119	105	0.3	217	208	1.2	677	589	0.3
Sex not known	0	0	0.0	1	0	0.0	2	0	0.0	2	0	0.0	5	0	0.0
Sex-site error	0	0	0.0	0	0	0.0	0	0	0.0	0	0	0.0	0	0	0.0
Site-morphology mismatch	98	98	0.2	77	77	0.2	49	49	0.1	26	26	<0.1	250	250	0.1
Age-site mismatch	0	0	0.0	0	0	0.0	0	0	0.0	0	0	0.0	0	0	0.0
Age-morphology mismatch	0	0	0.0	0	0	0.0	0	0	0.0	0	0	0.0	0	0	0.0
Age-site-morphology mismatch	0	0	0.0	0	0	0.0	0	0	0.0	0	0	0.0	0	0	0.0
Invalid dates**	6	5	<0.1	17	1	0.0	6	3	<0.1	2	0	0.0	31	9	<0.1
Death certificate only	1,799	1,769	5.8	1,156	1,147	3.3	788	780	1.8	381	381	1.4	4,124	4,077	2.4
Duplicate registration	0	0	0.0	0	0	0.0	0	0	0.0	0	0	0.0	0	0	0.0
Synchronous tumours†	220	186	0.9	139	108	0.2	102	84	0.2	52	43	0.2	498	406	0.2
Multiple primary same site‡	94	52	0.3	119	62	0.1	127	74	0.2	88	64	0.2	341	165	<0.1
**Total exclusions**		**2,243**	**5.1**		**1,538**	**3.4**		**1,095**	**2.2**		**722**	**2.3**		**5,598**	**3.3**
**Patients included in analysis**		**41,547**	**94.9**		**43,872**	**96.6**		**47,620**	**97.8**		**31,063**	**97.7**		**164,102**	**96.7**

* Missing year of birth; missing month and/or year of diagnosis; missing year of last known vital status

** For example, 31 February, or date of diagnosis earlier than date of birth

† Two or more invasive primary malignancies in the same person, in the same site, and with the same date of diagnosis

‡ Two or more invasive primary malignancies in the same person, in the same site, but with different dates of diagnosis

Web Appendix Table 4. Data quality control by calendar period of diagnosis (records and patients ineligible and excluded) - Lung cancer															
	1995-2000			2000-2004			2005-2009			2010-2012			1995-2012		
	Records	Patients	%	Records	Patients	%	Records	Patients	%	Records	Patients	%	Records	Patients	%
**Total registrered**	**162,160**	**161,897**	**100.0**	**37,347**	**37,314**	**100.0**	**33,470**	**33,410**	**100.0**	**18,851**	**18,807**	**100.0**	**132,038**	**131,868**	**100.0**
Incomplete data*	146	146	<0.1	49	49	<0.1	40	40	<0.1	25	25	<0.1	260	260	<0.1
Not resident in England	214	213	0.1	17	17	<0.1	5	5	<0.1	1	1	<0.1	237	236	<0.1
In situ neoplasm	289	274	0.2	343	318	0.2	363	309	0.2	237	217	0.2	1,232	1,118	0.2
Benign or uncertain	293	282	0.2	429	420	0.3	484	475	0.3	294	282	0.3	1,500	1,459	0.2
Metastatic	1,740	1,730	1.1	1,911	1,907	1.2	1,458	1,448	0.9	770	766	0.7	5,879	5,851	1.0
Kaposi Sarcoma	0	0	0.0	0	0	0.0	0	0	0.0	0	0	0.0	0	0	0.0
Otherwise ineligible	1,573	182	0.1	1,453	0	0.0	1,255	2	<0.1	752	2	<0.1	5,033	186	<0.1
Lymphoma	0	0	0.0	1	0	0.0	2	0	0.0	3	0	0.0	6	0	0.0
Leukaemia or myeloma	1	1	<0.1	0	0	0.0	0	0	0.0	0	0	0.0	1	1	<0.1
Aged 100+	14	13	<0.1	18	16	<0.1	44	39	<0.1	52	51	<0.1	128	119	<0.1
**Total ineligible**		**2,841**	**1.8**		**2,727**	**1.7**		**2,318**	**1.4**		**1,344**	**1.3**		**9,230**	**1.6**
**Total eligible**		**159,056**	**98.2**		**154,987**	**98.3**		**164,660**	**98.6**		**105,519**	**98.7**		**584,222**	**100.0**
Vital status unknown	395	217	0.1	386	325	0.2	179	134	<0.1	209	181	0.2	1,169	857	0.1
Sex not known	0	0	0.0	0	0	0.0	1	0	0.0	2	0	0.0	3	0	0.0
Sex-site error	0	0	0.0	0	0	0.0	0	0	0.0	0	0	0.0	0	0	0.0
Site-morphology mismatch	4	4	<0.1	4	4	<0.1	10	10	<0.1	1	1	<0.1	19	19	<0.1
Age-site mismatch	0	0	0.0	0	0	0.0	0	0	0.0	0	0	0.0	0	0	0.0
Age-morphology mismatch	0	0	0.0	0	0	0.0	0	0	0.0	0	0	0.0	0	0	0.0
Age-site-morphology mismatch	8	8	<0.1	4	4	<0.1	9	9	<0.1	8	8	<0.1	29	29	<0.1
Invalid dates**	25	12	<0.1	17	13	<0.1	16	10	<0.1	16	6	<0.1	74	41	<0.1
Death certificate only	14,908	14,763	9.3	9,309	9,223	6.0	7,978	7,866	4.8	2,951	2,910	2.8	35,146	34,762	6.0
Duplicate registration	0	0	0.0	0	0	0.0	0	0	0.0	0	0	0.0	0	0	0.0
Synchronous tumours†	758	683	0.4	645	466	0.3	745	534	0.3	496	359	0.3	2,644	2,042	0.3
Multiple primary same site‡	425	256	0.2	593	331	0.2	850	485	0.3	852	515	0.5	2,720	1,587	0.3
**Total exclusions**		**15,943**	**10.0**		**10,366**	**6.7**		**9,048**	**5.5**		**3,980**	**3.8**		**39,337**	**6.7**
**Patients included in analysis**		**143,113**	**90.0**		**144,621**	**93.3**		**155,612**	**94.5**		**101,539**	**96.2**		**544,885**	**93.3**

* Missing year of birth; missing month and/or year of diagnosis; missing year of last known vital status

** For example, 31 February, or date of diagnosis earlier than date of birth

† Two or more invasive primary malignancies in the same person, in the same site, and with the same date of diagnosis

‡ Two or more invasive primary malignancies in the same person, in the same site, but with different dates of diagnosis

Web Appendix Table 5. Data quality control by calendar period of diagnosis (records and patients ineligible and excluded) - Breast cancer															
	1995-2000			2000-2004			2005-2009			2010-2012			1995-2012		
	Records	Patients	%	Records	Patients	%	Records	Patients	%	Records	Patients	%	Records	Patients	%
**Total registrered**	**177,471**	**175,559**	**100.0**	**199,867**	**196,682**	**100.0**	**222,171**	**216,892**	**100.0**	**141,808**	**139,095**	**100.0**	**741,317**	**728,228**	**100.0**
Incomplete data*	204	204	0.1	44	43	<0.1	33	33	<0.1	17	17	<0.1	298	297	<0.1
Not resident in England	228	228	0.1	94	94	<0.1	6	6	<0.1	2	2	<0.1	330	330	<0.1
In situ neoplasm	11,128	10,579	6.0	16,708	15,930	8.1	23,134	21,333	9.8	15,252	14,325	10.3	66,222	62,167	8.5
Benign or uncertain	493	491	0.3	476	465	0.2	761	729	0.3	592	569	0.4	2,322	2,254	0.3
Metastatic	47	43	<0.1	43	43	<0.1	15	15	<0.1	1	1	<0.1	106	102	<0.1
Kaposi Sarcoma	0	0	0.0	0	0	0.0	0	0	0.0	0	0	0.0	0	0	0.0
Otherwise ineligible	0	0	0.0	0	0	0.0	0	0	0.0	0	0	0.0	0	0	0.0
Lymphoma	0	0	0.0	1	0	0.0	0	0	0.0	0	0	0.0	1	0	0.0
Leukaemia or myeloma	0	0	0.0	0	0	0.0	0	0	0.0	0	0	0.0	0	0	0.0
Aged 100+	126	126	<0.1	145	144	<0.1	176	173	<0.1	134	133	<0.1	581	576	<0.1
**Total ineligible**		**11,671**	**6.6**		**16,719**	**8.5**		**22,289**	**10.3**		**15,047**	**10.8**		**65,726**	**9.0**
**Total eligible**		**163,888**	**100.0**		**179,963**	**100.0**		**194,603**	**100.0**		**124,048**	**100.0**		**662,502**	**100.0**
Vital status unknown	705	504	0.3	662	614	0.3	437	410	0.2	688	673	0.5	2,492	2,201	0.3
Sex not known	0	0	0.0	0	0	0.0	0	0	0.0	0	0	0.0	0	0	0.0
Sex-site error	0	0	0.0	0	0	0.0	0	0	0.0	0	0	0.0	0	0	0.0
Site-morphology mismatch	29	29	<0.1	15	15	<0.1	15	15	<0.1	7	7	<0.1	66	66	<0.1
Age-site mismatch	10	10	<0.1	9	9	<0.1	13	13	<0.1	9	9	<0.1	41	41	<0.1
Age-morphology mismatch	0	0	0.0	0	0	0.0	0	0	0.0	0	0	0.0	0	0	0.0
Age-site-morphology mismatch	0	0	0.0	0	0	0.0	0	0	0.0	0	0	0.0	0	0	0.0
Invalid dates**	12	10	<0.1	72	7	<0.1	26	22	<0.1	4	3	<0.1	114	42	<0.1
Death certificate only	5,647	5,523	3.4	3,239	3,171	1.8	1,622	1,560	0.8	886	842	0.7	11,394	11,096	1.7
Duplicate registration	0	0	0.0	0	0	0.0	0	0	0.0	0	0	0.0	0	0	0.0
Synchronous tumours†	2,394	1,713	1.0	3,132	1,868	1.0	4,015	2,298	1.2	2,491	1,513	1.2	12,032	7,392	1.1
Multiple primary same site‡	3,791	2,743	1.7	5,924	4,127	2.3	8,670	6,038	3.1	6,002	4,686	3.8	24,387	17,594	2.7
**Total exclusions**		**10,532**	**6.4**		**9,811**	**5.5**		**10,356**	**5.3**		**7,733**	**6.2**		**38,432**	**5.8**
**Patients included in analysis**		**153,356**	**93.6**		**170,152**	**94.5**		**184,247**	**94.7**		**116,315**	**93.8**		**624,070**	**94.2**

* Missing year of birth; missing month and/or year of diagnosis; missing year of last known vital status

** For example, 31 February, or date of diagnosis earlier than date of birth

† Two or more invasive primary malignancies in the same person, in the same site, and with the same date of diagnosis

‡ Two or more invasive primary malignancies in the same person, in the same site, but with different dates of diagnosis

Web Appendix Table 6. Data quality control by calendar period of diagnosis (records and patients ineligible and excluded) - Ovarian cancer															
	**1995-2000**			**2000-2004**			**2005-2009**			**2010-2012**			**1995-2012**		
	**Records**	**Patients**	**%**	**Records**	**Patients**	**%**	**Records**	**Patients**	**%**	**Records**	**Patients**	**%**	**Records**	**Patients**	**%**
**Total registrered**	**30,352**	**30,278**	**100.0**	**31,669**	**31,590**	**100.0**	**32,592**	**32,493**	**100.0**	**20,220**	**20,183**	**100.0**	**114,833**	**114,544**	**0.0**
Incomplete data*	42	42	0.1	35	35	0.1	39	39	0.1	23	23	0.1	139	139	0.1
Not resident in England	39	39	0.1	9	9	<0.1	3	3	<0.1	1	1	<0.1	52	52	<0.1
In situ neoplasm	0	0	0.0	0	0	0.0	0	0	0.0	0	0	0.0	0	0	0.0
Benign or uncertain	1,045	1,029	3.4	1,130	1,087	3.4	1,634	1,615	5.0	945	930	4.6	4,754	4,661	4.1
Metastatic	169	165	0.5	194	194	0.6	119	119	0.4	56	56	0.3	538	534	0.5
Kaposi Sarcoma	0	0	0.0	0	0	0.0	0	0	0.0	0	0	0.0	0	0	0.0
Otherwise ineligible	111	6	<0.1	85	0	0.0	86	0	0.0	50	0	0.0	332	6	<0.1
Lymphoma	0	0	0.0	0	0	0.0	0	0	0.0	0	0	0.0	0	0	0.0
Leukaemia or myeloma	0	0	0.0	0	0	0.0	0	0	0.0	0	0	0.0	0	0	0.0
Aged 100+	6	6	<0.1	12	12	<0.1	13	13	<0.1	6	6	<0.1	37	37	<0.1
**Total ineligible**		**1,287**	**4.3**		**1,337**	**4.2**		**1,789**	**5.5**		**1,016**	**5.0**		**5,429**	**4.7**
**Total eligible**		**28,991**	**100.0**		**30,253**	**100.0**		**30,704**	**100.0**		**19,167**	**100.0**		**109,115**	**100.0**
Vital status unknown	98	54	0.2	73	65	0.2	86	58	0.2	65	55	0.3	322	232	0.2
Sex not known	0	0	0.0	0	0	0.0	0	0	0.0	0	0	0.0	0	0	0.0
Sex-site error	0	0	0.0	0	0	0.0	0	0	0.0	0	0	0.0	0	0	0.0
Site-morphology mismatch	2,330	2,330	8.0	2,914	2,914	9.6	3,387	3,387	11.0	2,444	2,444	12.8	11,075	11,075	10.1
Age-site mismatch	0	0	0.0	0	0	0.0	0	0	0.0	0	0	0.0	0	0	0.0
Age-morphology mismatch	0	0	0.0	0	0	0.0	0	0	0.0	0	0	0.0	0	0	0.0
Age-site-morphology mismatch	0	0	0.0	0	0	0.0	0	0	0.0	0	0	0.0	0	0	0.0
Invalid dates**	2	1	<0.1	4	2	<0.1	4	1	<0.1	1	0	0.0	11	4	<0.1
Death certificate only	1,746	1,717	5.9	1,186	1,176	3.9	742	737	2.4	375	371	1.9	4,049	4,001	3.7
Duplicate registration	0	0	0.0	0	0	0.0	0	0	0.0	0	0	0.0	0	0	0.0
Synchronous tumours†	107	75	0.3	108	71	0.2	128	81	0.3	54	33	0.2	397	260	0.2
Multiple primary same site‡	82	43	0.1	114	70	0.2	174	104	0.3	97	70	0.4	467	287	0.3
**Total exclusions**		**4,220**	**14.6**		**4,298**	**14.2**		**4,368**	**14.2**		**2,973**	**15.5**		**15,859**	**14.5**
**Patients included in analysis**		**24,771**	**85.4**		**25,955**	**85.8**		**26,336**	**85.8**		**16,194**	**84.5**		**93,256**	**85.5**

*** Missing year of birth; missing month and/or year of diagnosis; missing year of last known vital status**

** For example, 31 February, or date of diagnosis earlier than date of birth

† Two or more invasive primary malignancies in the same person, in the same site, and with the same date of diagnosis

‡ Two or more invasive primary malignancies in the same person, in the same site, but with different dates of diagnosis

Web Appendix Table 7. Arithmetic gap in one-year net survival with 95% confidence intervals for adults (aged 15-99 years) between England and each other country, by calendar period of diagnosis, country and cancer site

	1995-1999	2000-2004	2005-2009
**Stomach**			
Australia	15.0 (13.8, 16.3)	13.2 (12.0, 14.5)	11.6 (10.2, 12.9)
Canada	8.1 (7.1, 9.1)	6.0 (5.0, 7.0)	4.6 (3.5, 5.6)
Denmark	-0.6 (-2.6, 1.3)	-3.9 (-5.9, -1.9)	-0.9 (-2.9, 1.0)
Norway	7.1 (5.1, 9.1)	6.4 (4.3, 8.5)	5.9 (3.7, 8.0)
Sweden	7.2 (5.7, 8.7)	4.8 (3.2, 6.4)	5.2 (3.5, 6.9)
**Colon**			
Australia	10.5 (9.9, 11.0)	10.7 (10.2, 11.2)	10.5 (10.0, 11.0)
Canada	7.7 (7.2, 8.2)	8.2 (7.7, 8.6)	7.7 (7.3, 8.1)
Denmark	0.8 (-0.2, 1.8)	0.5 (-0.4, 1.4)	1.5 (0.6, 2.3)
Norway	7.3 (6.3, 8.3)	5.9 (5.0, 6.8)	5.9 (5.1, 6.8)
Sweden	8.4 (7.5, 9.2)	8.2 (7.4, 9.0)	8.4 (7.7, 9.1)
**Rectum**			
Australia	8.9 (8.1, 9.7)	7.7 (7.0, 8.5)	6.9 (6.2, 7.6)
Canada	7.1 (6.3, 8.0)	5.0 (4.2, 5.8)	4.6 (4.0, 5.3)
Denmark	-0.4 (-1.7, 0.9)	0.2 (-0.9, 1.3)	1.5 (0.5, 2.5)
Norway	6.8 (5.5, 8.0)	4.3 (3.2, 5.5)	5.4 (4.3, 6.4)
Sweden	7.6 (6.6, 8.5)	5.4 (4.5, 6.2)	4.4 (3.6, 5.3)
**Lung**			
Australia	13.9 (13.3, 14.5)	12.6 (12, 13.2)	10.9 (10.3, 11.6)
Canada	12.2 (11.8, 12.6)	9.6 (9.1, 10.0)	8.4 (8.0, 8.8)
Denmark	1.1 (0.3, 1.8)	3.2 (2.5, 4.0)	4.0 (3.2, 4.8)
Norway	6.7 (5.7, 7.7)	4.6 (3.6, 5.6)	8.0 (7.0, 9.0)
Sweden	9.4 (8.5, 10.3)	8.8 (8.0, 9.7)	9.4 (8.6, 10.3)
**Breast**			
Australia	5.5 (5.2, 5.9)	3.8 (3.5, 4.1)	2.4 (2.1, 2.7)
Canada	4.7 (4.4, 5.0)	2.8 (2.6, 3.1)	1.4 (1.1, 1.6)
Denmark	2.2 (1.6, 2.8)	1.5 (1.0, 2.0)	1.0 (0.6, 1.5)
Norway	4.8 (4.2, 5.4)	3.1 (2.5, 3.6)	2.2 (1.7, 2.7)
Sweden	5.8 (5.4, 6.2)	4.1 (3.7, 4.4)	2.9 (2.6, 3.2)
**Ovary**			
Australia	13.6 (12.1, 15.0)	12.8 (11.5, 14.1)	10.6 (9.3, 12.0)
Canada	10.9 (9.6, 12.2)	8.6 (7.3, 9.8)	7.4 (6.3, 8.6)
Denmark	4.4 (2.6, 6.2)	5.4 (3.7, 7.2)	6.5 (4.8, 8.3)
Norway	11.8 (9.8, 13.7)	13.3 (11.5, 15.1)	10.6 (8.8, 12.4)
Sweden	18.3 (16.8, 19.7)	16.5 (15.0, 17.9)	15.4 (14.0, 16.8)

The gap was calculated by subtracting net survival in England from that in each other country, and is expressed as the arithmetic difference between the two percentages. Confidence intervals were calculated using the formula for the confidence interval for the difference between two means with normal distributions.

Web Appendix Table 8. Arithmetic gap in five-year net survival with 95% confidence intervals for adults (aged 15-99 years) between England and each other country, by calendar period of diagnosis, country and cancer site

	1995-1999	2000-2004	2005-2009
**Stomach**			
Australia	11.6 (10.4, 12.8)	11.5 (10.3, 12.6)	9.7 (8.4, 11.0)
Canada	6.8 (5.9, 7.7)	6.8 (5.8, 7.7)	6.6 (5.7, 7.6)
Denmark	-0.5 (-2.1, 1.1)	-1.0 (-2.7, 0.6)	-0.3 (-2.0, 1.5)
Norway	6.8 (5.0, 8.6)	5.7 (3.7, 7.6)	5.9 (3.8, 8.0)
Sweden	6.9 (5.5, 8.3)	4.7 (3.2, 6.1)	5.0 (3.5, 6.6)
**Colon**			
Australia	12.8 (12.0, 13.5)	12.2 (11.4, 12.9)	10.5 (9.8, 11.2)
Canada	9.3 (8.6, 9.9)	9.2 (8.5, 9.8)	9.1 (8.5, 9.7)
Denmark	0.7 (-0.6, 1.9)	1.2 (-0.1, 2.4)	2.2 (1.0, 3.3)
Norway	8.4 (7.0, 9.7)	7.5 (6.2, 8.7)	8.1 (6.9, 9.3)
Sweden	7.9 (6.7, 9.0)	8.5 (7.4, 9.5)	8.8 (7.8, 9.8)
**Rectum**			
Australia	11.1 (9.9, 12.4)	10.0 (8.9, 11.0)	7.9 (6.9, 9.0)
Canada	7.7 (6.5, 9.0)	6.7 (5.5, 7.8)	6.5 (5.5, 7.6)
Denmark	-1.2 (-2.9, 0.6)	0.0 (-1.7, 1.6)	2.1 (0.6, 3.7)
Norway	9.0 (7.2, 10.9)	7.9 (6.1, 9.6)	8.3 (6.6, 10.1)
Sweden	9.1 (7.7, 10.6)	5.8 (4.4, 7.1)	5.7 (4.4, 7.0)
**Lung**			
Australia	6.7 (6.2, 7.1)	6.4 (6.0, 6.8)	5.5 (5.0, 6.0)
Canada	8.1 (7.8, 8.4)	7.2 (6.9, 7.5)	7.8 (7.5, 8.1)
Denmark	1.0 (0.4, 1.5)	1.2 (0.7, 1.7)	1.8 (1.2, 2.5)
Norway	3.7 (2.9, 4.4)	3.3 (2.5, 4.1)	5.5 (4.7, 6.4)
Sweden	5.2 (4.5, 5.8)	4.9 (4.2, 5.6)	6.1 (5.4, 6.9)
**Breast**			
Australia	10.7 (10.0, 11.4)	7.8 (7.2, 8.4)	5.1 (4.4, 5.8)
Canada	9.8 (9.2, 10.3)	6.7 (6.1, 7.2)	4.7 (4.3, 5.2)
Denmark	1.9 (0.8, 2.9)	2.1 (1.1, 3.0)	0.9 (0.0, 1.9)
Norway	7.6 (6.4, 8.8)	5.5 (4.4, 6.6)	4.8 (3.7, 5.9)
Sweden	9.9 (9.1, 10.7)	7.0 (6.2, 7.7)	5.1 (4.4, 5.9)
**Ovary**			
Australia	7.9 (6.4, 9.4)	7.4 (5.9, 8.9)	6.0 (4.4, 7.5)
Canada	8.3 (6.9, 9.7)	5.9 (4.5, 7.2)	6.0 (4.6, 7.4)
Denmark	3.0 (1.1, 4.9)	3.6 (1.7, 5.4)	5.8 (3.7, 7.8)
Norway	8.5 (6.3, 10.7)	10.6 (8.4, 12.8)	8.8 (6.6, 11.0)
Sweden	12.6 (10.8, 14.4)	13.2 (11.4, 14.9)	12.0 (10.2, 13.7)

The gap was calculated by subtracting net survival in England from that in each other country, and is expressed as the arithmetic difference between the two percentages. Confidence intervals were calculated using the formula for the confidence interval for the difference between two means with normal distributions.
